# Global Burden of Hepatoblastoma From 1990 to 2021 and Projection to 2030

**DOI:** 10.1002/cam4.71163

**Published:** 2025-08-19

**Authors:** Xiaoyu Huang, Zhiqiang Zhang, Qiong Wu, Shaoyang Kang, Xinyue Yang, Fengmei Wang

**Affiliations:** ^1^ School of Medicine Nankai University Tianjin China; ^2^ Department of Gastroenterology and Hepatology, Tianjin Key Laboratory of Molecular Diagnosis and Treatment of Liver Cancer Tianjin First Central Hospital, Nankai University Tianjin China; ^3^ Graduate School of Tianjin Medical University, Tianjin Medical University Tianjin China

**Keywords:** Bayesian age‐period‐cohort modeling, global burden, hepatoblastoma, pediatric population, prediction

## Abstract

**Background:**

Hepatoblastoma (HB) is the most common primary pediatric hepatocellular carcinoma, and the five‐year survival rate for HB is the lowest among childhood cancers, as 20% of cases are chemotherapy resistant or unresectable. Understanding the global burden of HB and future trends in HB burden is urgently needed for gastroenterology and hepatology specialists and pediatricians. This study aims to provide a comprehensive assessment of the global burden of hepatoblastoma, predict future trends over the next decade, and offer insights into its management.

**Methods:**

Using data from the Global Burden of Disease 2021, trends in HB‐related mortality and disability‐adjusted life years (DALYs) were analyzed. Age‐standardized rates (ASR) and estimated annual percentage changes (EAPC) were employed to measure trends in these outcomes. Joinpoint regression identified trends, while Bayesian age‐period‐cohort (BAPC) modeling projected the future burden.

**Results:**

Although the global number of HB‐related deaths decreased from 1990 to 2021, the number of deaths in 2021 in low socio‐demographic index (SDI) regions remained comparable to 1990 levels, with a notable upward trend in 27 countries, particularly in West Africa. Mortality and DALYs rates were highest in the early neonatal period, with the 2–4 years age group also showing the greatest burden. Projections indicate a global decline in HB burden from 2021 to 2030, but regions such as West Africa are likely to face persistent challenges.

**Conclusions:**

In light of rapid population growth and escalating poverty in Africa, early screening and intervention for hepatoblastoma, particularly among children under five in low SDI regions, are critical to reducing the disease burden. These findings have significant implications for liver health policy, emphasizing the need for targeted interventions based on age and region to mitigate the impact of hepatoblastoma.

**Abbreviations**
AAPC: annual average percentage change
APC: annual percentage change
ASDR: age‐standardized disability rates
ASMR: age‐standardized mortality rates
ASR: Age‐standardized rates
BAPC: Bayesian age‐period‐cohort
Cis: confidence intervals
DALYs: disability‐adjusted life years
EAPC: estimated annual percentage change
EAPC: estimated annual percentage changes
GBD: Global Burden of Disease
HB: hepatoblastoma
HCC: hepatocellular carcinoma
INLA: integrated nested laplace approximation
LOESS: locally estimated scatterplot smoothing
NASH: non‐alcoholic steatohepatitis
SDI: socio‐demographic index
WHO: World Health Organization

## 1 Background

Hepatoblastoma (HB) is the most common primary liver cancer in children [1]. The standard treatment consists of surgical resection and chemotherapy; while the prognosis for patients with resectable tumors is generally favorable, outcomes for those with non‐resectable or recurrent disease remain poor [2]. Approximately 20% of HB cases are resistant to chemotherapy or unresectable, contributing to the lowest five‐year survival rate among childhood cancers [3]. Global burden of disease data from 1990 to 2021 shows a decline in the number of people affected by HB, from 56,183.04 in 1990 to 33,829.16 in 2021. During the same period, HB‐related deaths decreased from 4828.29 in 1990 to 2416.17 in 2021, accounting for 0.03% of all global deaths. Notably, nearly 70% of these deaths occur in low and low‐middle‐SDI regions, where low‐income levels and high fertility rates further accelerate the development of these diseases [4]. Given that the majority of the global population resides in these regions, urgent measures to control the progression of HB are needed.

While previous studies have identified the global burden of disease for hepatocellular carcinoma and quantified mortality and disability‐adjusted life years (DALYs) for other types of hepatocellular carcinoma [5], such as hepatitis B‐induced hepatocellular carcinoma [6], hepatitis C‐to‐hepatocellular carcinoma [7], alcoholic liver cancer [8], and hepatocellular carcinoma due to NASH [9], no studies have examined trends in the burden of hepatoblastoma by sex and SDI region or projected its global burden through 2030. The Global Burden of Disease (GBD) database, maintained by the Institute for Health Metrics and Evaluation, offers comprehensive data on diseases, injuries, and health risk factors globally, facilitating comparisons by time, sex, age, and region [10].

Using data from GBD 2021, we analyzed the global, regional, and national burdens of HB. The risk of HB deaths varies not only with regional SDIs but also with advances in diagnostic and treatment technologies. Early diagnosis and intervention significantly impact HB outcomes, and regional differences in SDI contribute to disparities in mortality rates. In addition, changes in national policies and economic investments in HB disease management affect HB mortality and the DALYs rate. This study provides an in‐depth analysis of trends in HB mortality and DALYs rate over time, highlighting the strengths and weaknesses of current health policies to guide the allocation of healthcare resources.

## 2 Methods

### 2.1 Data Source

This study primarily used data from the Global Burden of Disease (GBD) 2021 database, which reports the burden of 369 diseases and injuries across 204 countries and 21 regions from 1990 to 2021, with a focus on age, gender, and region‐specific demographics [11]. This approach integrates existing data to compensate for incomplete healthcare information, enabling estimation of disease burden across different regions. Ethical considerations for this study adhered to the principles of the Declaration of Helsinki, and the University of Washington's Institutional Review Board waived the requirement for informed consent due to the use of de‐identified aggregated data. The study followed the guidelines of the GATHER checklist for accurate and transparent health estimates [12].

### 2.2 Disease Burden Indicators

Common indicators for assessing disease burden include mortality and disability‐adjusted life years (DALYs). This study aims to analyze global trends in HB mortality and DALYs rate from 1990 to 2021, with projections through 2030. Mortality measures include the number of deaths, crude death rates for all age groups, age‐standardized mortality rates (ASMR), relative percentage changes, and 95% confidence intervals (CIs). DALYs rate measures include DALYs for all age groups, age‐standardized disability rates (ASDR), relative percentage changes, and 95% CIs. The analysis also includes the Socio‐Demographic Index (SDI), a composite indicator reflecting a country's development level based on per capita income, average years of schooling, and fertility rates for women under 25 years [13]. SDI values range from 0 to 1, with higher values indicating a greater level of socio‐economic development. All rates are presented per 100,000 population, with 95% uncertainty intervals (UIs) derived from the 2.5th and 97.5th posterior distribution values for an ordered sample of 1000 based on the GBD algorithm.

### 2.3 Statistical Analysis

We analyzed the relationship between age‐standardized mortality rate (ASMR) or age‐standardized disability rate (ASDR) and SDI using a Gaussian process regression model and a locally estimated scatterplot smoothing (LOESS) smoother, respectively. This relationship was assessed by the Spearman rank correlation test. Uncertainty intervals (UI) of 95% were reported for all indicators, and all rates are shown per 100,000 inhabitants. Joinpoint regression was used to analyze the annual percentage change (APC) in age‐standardized HB mortality rates and DALYs for different time intervals between 1990 and 2021, as well as the overall annual average percentage change (AAPC). This approach helps to understand trends and turning points in mortality, which is essential for identifying the causes of changes in mortality and focusing on relevant interventions.

Joinpoint regression analysis was performed using the Joinpoint software version 5.1.0.0 (National Cancer Institute). The analysis was configured to allow a maximum of 3 joinpoints, with a minimum of 4 years between joinpoints. The optimal number of joinpoints was determined using the Monte Carlo permutation method with 5000 iterations, selecting the model with the best fit while avoiding overfitting based on the Bayesian Information Criterion (BIC).

The Bayesian Age‐Period‐Cohort (BAPC) model has been validated for projecting various health outcomes beyond mortality, including incidence and DALYs, particularly in rare disease studies. While originally developed for mortality projections, the BAPC model's strength lies in its ability to account for age, period, and cohort effects simultaneously, making it suitable for DALY projections when the underlying epidemiological patterns follow similar temporal trends as mortality.

Finally, we employed Bayesian age‐period‐cohort (BAPC) modeling to project global burden trends for HB from 2021 to 2030 [14, 15, 16, 17, 18]. The BAPC model assumes separable age, period, and cohort effects following log‐linear structures. Based on the assumption that the effects of age, period, and cohort are similar in time, Bayesian inference utilizes second‐order stochastic offsets in the BAPC model, smooths the three aforementioned prior values, and predicts posterior rates. Prior distributions included weakly informative normal priors for age effects and second‐order random walk priors for period and cohort effects with Gamma (1, 0.00005) precision parameters. Population data were obtained from the World Health Organization (WHO). BAPC employs the Integrated Nested Laplace Approximation (INLA) to approximate the marginal posterior distributions. INLA approximates the marginal posterior distributions using a combination of Laplace approximations and numerical integration, providing credible intervals that account for both parameter uncertainty and model uncertainty. The width of uncertainty intervals reflects data quality and availability—wider intervals typically indicate regions with sparse data or higher epidemiological uncertainty. The software packages employed in this study included “BAPC” and “INLA”. Statistical significance was set at *p* < 0.05.

Statistical significance for joinpoint selection was set at *p* < 0.05. Statistical significance of the parameters was assessed using a chi‐square test, and the significance level was set at 0.05. Data were analyzed using R software version 4.3.2, and trends were analyzed using Joinpoint software version 5.1.0.0 [19].

## 3 Results

### 3.1 Global, Regional and National Trends in Burden of Hepatoblastoma

In 2021, HB global deaths were approximately 2416.17 per 100,000 (95% UI: 1922.47–3019.03). Over the past 32 years, the global number of HB deaths decreased by 49.96% (95% UI: 30.65%–60.73%). The changes in all‐age HB mortality and age‐standardized mortality rates (ASMR) exhibited similar trends (Table 1). A corresponding reduction was observed in disability‐adjusted life years (DALYs) for HB, with the global DALYs rate amounting to 213,477.91 per 100,000 (95% UI: 170,089.51–267,250.26) in 2021. This reflects a 49.93% reduction (95% UI: 30.66%–60.74%) in global HB DALYs over the past three decades. Both the all‐age DALYs rate and the age‐standardized disability rate (ASDR) demonstrated similar trends (Table 2).

**TABLE 1:** The trends of burden in death from hepatoblastoma by sex and region, 1990–2021.

Characteristics	Deaths		All‐age mortality		Age‐standardized mortality	
	Number in 2021, *n*	Change of numbers 1990–2021, %	Rate in 2021, per 100,000	Percent change 1990–2021, %	Rate in 2021, per 100,000	Percent change 1990–2021, %
**Global**						
Male	1218.56 (979.64–1519.52)	−52.18 (−62.05 to −35.8)	0.03 (0.02 to 0.04)	−67.56 (−74.26 to −56.45)	0.04 (0.03–0.04)	−54.92 (−64.27 to −39.24)
Female	1197.61 (929.46–1537.48)	−47.48 (−61.24 to −24.05)	0.03(0.02–0.04)	−64.63 (−73.9 to −48.86)	0.04 (0.03–0.05)	−50.06 (−63.22 to −27.73)
Both	2416.17 (1922.47–3019.03)	−49.96 (−60.73 to −30.65)	0.03 (0.02–0.04)	−66.18 (−73.46 to −53.13)	0.04 (0.03–0.05)	−52.63 (−62.85 to −34.36)
**Low SDI**						
Male	451.13 (322.67–608.43)	−1.76 (−30.67 to 71.04)	0.08 (0.06–0.11)	−55.66 (−68.71 to −22.81)	0.05 (0.04–0.07)	−46.35 (−62.25 to −5.37)
Female	536.25 (366.4–773.29)	−3.06 (−35.37 to 53.15)	0.1 (0.07–0.14)	−56.77 (−71.18 to −31.7)	0.07 (0.05–0.1)	−46.25 (−64.4 to −13.62)
Both	987.38 (696.1–1339.07)	−2.47 (−31.35 to 54.72)	0.09 (0.06–0.12)	−56.24 (−69.2 to −30.59)	0.06 (0.04–0.08)	−46.31 (−62.4 to −13.35)
**Low‐middle SDI**						
Male	379.69 (301.67–484.83)	−37.72 (−54.88 to 0.29)	0.04 (0.03–0.05)	−61.87 (−72.38 to −38.6)	0.04 (0.03–0.05)	−43.77 (−59.31 to −9.05)
Female	372.6 (297.22–450.16)	−33.14 (−54.55 to 7.4)	0.04 (0.03–0.05)	−60.1 (−72.88 to −35.9)	0.04 (0.03–0.05)	−39.29 (−58.75 to −2.06)
Both	752.3 (603.54–919.55)	−35.53 (−52.03 to 1.42)	0.04 (0.03–0.05)	−61.03 (−71 to −38.69)	0.04 (0.03–0.05)	−41.63 (−56.56 to −7.94)
**Middle SDI**						
Male	259.38 (205.14–327.22)	−74.73 (−80.45 to −66.23)	0.02 (0.02–0.03)	−82.03 (−86.09 to −75.98)	0.03 (0.02–0.04)	−71.71 (−78.17 to −62.1)
Female	199.49 (161.27–249.27)	−75.54 (−81.88 to −65.68)	0.02 (0.01–0.02)	−82.98 (−87.39 to −76.12)	0.02 (0.02–0.03)	−72.15 (−79.5 to −60.7)
Both	458.87 (369.31–569.08)	−75.09 (−80.38 to −67.19)	0.02 (0.02–0.02)	−82.47 (−86.19 to −76.92)	0.03 (0.02–0.03)	−71.89 (−77.91 to −62.87)
**High‐middle SDI**						
Male	76.43 (62.02–93.93)	−79.04 (−83.16 to −73.22)	0.01 (0.01–0.01)	−83 (−86.34 to −78.28)	0.02 (0.02–0.03)	−72.83 (−78.27 to −65.34)
Female	54.87 (45.03–68.26)	−81.44 (−86.25 to −75.16)	0.01 (0.01–0.01)	−84.78 (−88.72 to −79.63)	0.02 (0.01–0.02)	−75.43 (−81.99 to −66.56)
Both	131.3 (108.76–158.63)	−80.12 (−84.27 to −75.05)	0.01 (0.01–0.01)	−83.78 (−87.17 to −79.65)	0.02 (0.02–0.02)	−73.96 (−79.54 to −67.07)
**High SDI**						
Male	51.35 (46.37–55.97)	−40.8 (−47.62 to −33.29)	0.01 (0.01–0.01)	−52.98 (−58.4 to −47.02)	0.02 (0.02–0.02)	−32.28 (−40.16 to −23.65)
Female	33.86 (31.53–36.06)	−41.24 (−47.97 to −34.06)	0.01 (0.01–0.01)	−52.2 (−57.67 to −46.35)	0.01 (0.01–0.01)	−32.7 (−40.44 to −24.58)
Both	85.22 (78.12–91.64)	−40.98 (−46.47 to −34.94)	0.01 (0.01–0.01)	−52.55 (−56.97 to −47.7)	0.02 (0.01–0.02)	−32.45 (−38.83 to −25.46)

**TABLE 2:** The trends of burden in DALYs from hepatoblastoma by sex and region, 1990–2021.

Characteristics	Dalys		All‐age mortality		Age‐standardized mortality	
	Number in 2021, *n*	Change of numbers 1990–2021, %	Rate in 2021, per 100,000	Percent change 1990–2021, %	Rate in 2021, per 100,000	Percent change 1990–2021, %
**Global**						
Male	107615.01 (86,429–134376.39)	−52.11 (−62 to −35.65)	2.72 (2.18–3.39)	−67.52 (−74.23 to −56.35)	3.19 (2.56–3.97)	−54.78 (−64.18 to −38.99)
Female	105862.9 (82257.59–135573.72)	−47.5 (−61.28 to −24.12)	2.69 (2.09–3.45)	−64.64 (−73.92 to −48.9)	3.36 (2.61–4.31)	−50 (−63.2 to −27.68)
Both	213477.91 (170089.51–267250.26)	−49.93 (−60.74 to −30.66)	2.71 (2.16–3.39)	−66.16 (−73.47 to −53.14)	3.27 (2.61–4.1)	−52.52 (−62.8 to −34.25)
**Low SDI**						
Male	39965.9 (28641.74–53993.9)	−1.64 (−30.57 to 70.68)	7.15 (5.12–9.66)	−55.61 (−68.67 to −22.97)	4.71 (3.37–6.37)	−46.22 (−62.15 to −5.35)
Female	47539.42 (32444.58–68442.26)	−3.11 (−35.33 to 52.64)	8.52 (5.81–12.26)	−56.79 (−71.16 to −31.93)	5.85 (3.99–8.43)	−46.21 (−64.38 to −13.96)
Both	87505.32 (61642.88–118845.84)	−2.45 (−31.33 to 54.23)	7.83 (5.52–10.64)	−56.23 (−69.19 to −30.81)	5.27 (3.71–7.16)	−46.23 (−62.34 to −13.51)
**Low‐middle SDI**						
Male	33518.1 (26610.87–42859.87)	−37.69 (−54.86 to 0.22)	3.47 (2.76–4.44)	−61.85 (−72.36 to −38.64)	3.4 (2.7–4.35)	−43.66 (−59.23 to −8.96)
Female	32874.78 (26278.62–39755.8)	−33.15 (−54.56 to 7.19)	3.44 (2.75–4.16)	−60.11 (−72.88 to −36.03)	3.55 (2.84–4.3)	−39.22 (−58.71 to −2.04)
Both	66392.88 (53247.05–80992.05)	−35.52 (−52 to 1.26)	3.46 (2.77–4.22)	−61.02 (−70.98 to −38.78)	3.47 (2.78–4.24)	−41.54 (−56.48 to −7.94)
**Middle SDI**						
Male	22763.97 (18002.48–28769.11)	−74.83 (−80.56 to −66.31)	1.85 (1.46–2.33)	−82.1 (−86.18 to −76.04)	2.48 (1.95–3.14)	−71.72 (−78.22 to −62.1)
Female	17535.02 (14166.03–22009.23)	−75.68 (−82.06 to −65.74)	1.44 (1.17–1.81)	−83.07 (−87.51 to −76.16)	2.09 (1.68–2.63)	−72.22 (−79.63 to −60.71)
Both	40298.99 (32391.32–50165.4)	−75.21 (−80.51 to −67.3)	1.65 (1.32–2.05)	−82.55 (−86.28 to −76.99)	2.29 (1.84–2.86)	−71.93 (−77.97 to −62.87)
**High‐middle SDI**						
Male	6738.42 (5467.9–8278.06)	−79.02 (−83.17 to −73.2)	1.03 (0.84–1.27)	−82.98 (−86.35 to −78.26)	1.84 (1.49–2.28)	−72.66 (−78.17 to −65.14)
Female	4841.56 (3962.98–6009.5)	−81.46 (−86.29 to −75.02)	0.74 (0.61–0.92)	−84.79 (−88.76 to −79.51)	1.47 (1.2–1.82)	−75.35 (−81.97 to −66.29)
Both	11579.98 (9596.84–13953.73)	−80.11 (−84.34 to −74.88)	0.89 (0.74–1.07)	−83.78 (−87.22 to −79.51)	1.66 (1.37–2.01)	−73.83 (−79.5 to −66.83)
**High SDI**						
Male	4577.78 (4138.52–4985.37)	−40.16 (−46.9 to −32.56)	0.84 (0.76–0.91)	−52.48 (−57.83 to −46.43)	1.65 (1.49–1.79)	−31.43 (−39.23 to −22.64)
Female	3025.9 (2815.56–3224.94)	−40.62 (−47.31 to −33.36)	0.55 (0.51–0.59)	−51.69 (−57.13 to −45.79)	1.15 (1.07–1.23)	−31.86 (−39.58 to −23.59)
Both	7603.68 (6978.67–8189.41)	−40.35 (−45.86 to −34.21)	0.7 (0.64–0.75)	−52.04 (−56.48 to −47.11)	1.41 (1.29–1.52)	−31.61 (−37.86 to −24.61)

**FIGURE 1:**
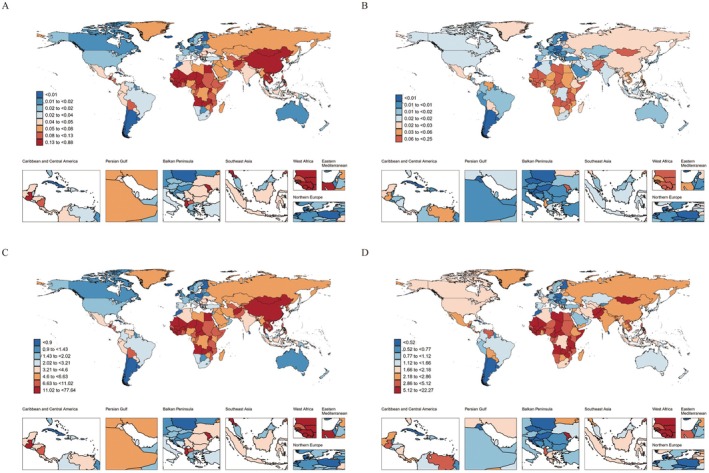
Global Distribution of ASMR and ASDR for Hepatoblastoma in 1990 and 2021. Global distribution of ASMR for hepatoblastoma in 1990 (A) and 2021 (B), and global distribution of ASDR attributable to hepatoblastoma in 1990 (C) and 2021 (D).

Geographic disparities were also evident, with West Africa consistently exhibiting the highest ASMR for HB, both in 1990 and 2021. The rates were significantly higher than those observed in the Nordic region during the same period (Figure 1A,B). A similar pattern was observed for ASDR across these regions (Figure 1C,D).

At the national level, in 1990, Mongolia had the highest HB mortality rate, with 0.88 deaths per 100,000 (95% UI: 0.54–1.37), followed by Guinea and Mali (Table S1). In 2021, Mali recorded the highest rate, with 0.25 per 100,000 (95% UI: 0.15–0.38), followed by Gambia and Guinea (Table S2). Overall, ASMR declined in 197 countries, with an increase in 27 countries (Tables S1 and S2). A similar pattern was observed for ASDR. In 1990, Mongolia had the highest HB DALYs rate, at 77.64 per 100,000 (95% UI: 47.05–120.78), followed by Guinea and Mali (Table S3). By 2021, Mali had the highest DALY rate at 22.26 (95% UI: 13.04–33.42) per 100,000, with Gambia and Guinea following (Table S4). Overall, ASDR decreased in 197 countries, with an increase in 27 countries (Tables S3 and S4).

### 3.2 Trends in the Burden of Hepatoblastoma by SDI and Gender

There are notable disparities in the burden of hepatoblastoma (HB) across different socio‐demographic index (SDI) regions. Although the global number of HB‐related deaths decreased by 49.96% (95% UI: 30.65%–60.73%) over the last 30 years, reaching 2416.17 per 100,000 population in 2021, there is considerable variation in mortality trends across SDI regions. The high SDI group exhibited the lowest mortality rate at 85.22 (95% UI: 78.12–91.64) per 100,000, marking a 40.98% reduction (95% UI: 34.94%–46.47%) since 1990. In contrast, the low SDI group had the highest mortality rate at 987.38 (95% UI: 696.1–1339.07) per 100,000, with a minimal reduction of 2.47% (95% UI: −54.72% to 31.35%) since 1990 (Table 1). Despite a global decline in HB‐related deaths from 1990 to 2021, the mortality rate in low‐SDI regions has remained largely unchanged since 1990 (Table 1). The burden of DALYs associated with HB mirrors mortality trends. Although the global number of DALYs for HB decreased by 49.93% (95% UI: 30.66%–60.74%) over the past three decades, reaching 2,134,777.91 per 100,000 in 2021 (95% UI: 1,700,089.51–2,672,250.26), substantial variation exists across SDI regions. The high SDI group had the lowest DALY rate at 7603.68 (95% UI: 6978.67–8189.41) per 100,000, a decrease of 40.35% (95% UI: 34.21%–45.86%) from 1990. In contrast, the low SDI group had the highest DALYs rate at 87,505.32 (95% UI: 61,642.88–118,845.84) per 100,000, with a negligible decrease of 2.45% (95% UI: −54.23% to 31.33%) since 1990 (Table 2). Overall, while the global DALY burden for HB has continued to decline, the DALY rate in low‐SDI regions has changed little since 1990.

In the sex‐disaggregated analysis, global deaths decreased for both males and females. In 2021, male deaths were 1218.56 (95% UI: 979.64–1519.52); female deaths were 1,197.61 (95% UI: 929.46–1537.48) in 2021, representing a 52.18% decrease (95% UI: 35.8%–62.05%) and a 47.48% decrease (95% UI: 24.05%–61.24%) compared to 1990, respectively (Table 1). A similar trend was seen in DALYs, with males at 107,615.01 (95% UI: 86,429–134,376.39) and females at 105,862.9 (95% UI: 82,257.59–135,573.72) in 2021, representing a 52.11% decrease (95% UI: 35.65%–62%) and a 47.5% decrease (95% UI: 24.12%–61.28%) since 1990 (Table 2). Overall, male deaths and DALYs were higher. However, in low‐SDI regions, females had a higher burden (Tables 1 and 2).

### 3.3 Global Trends in Hepatoblastoma Across Different Age Groups

**FIGURE 2:**
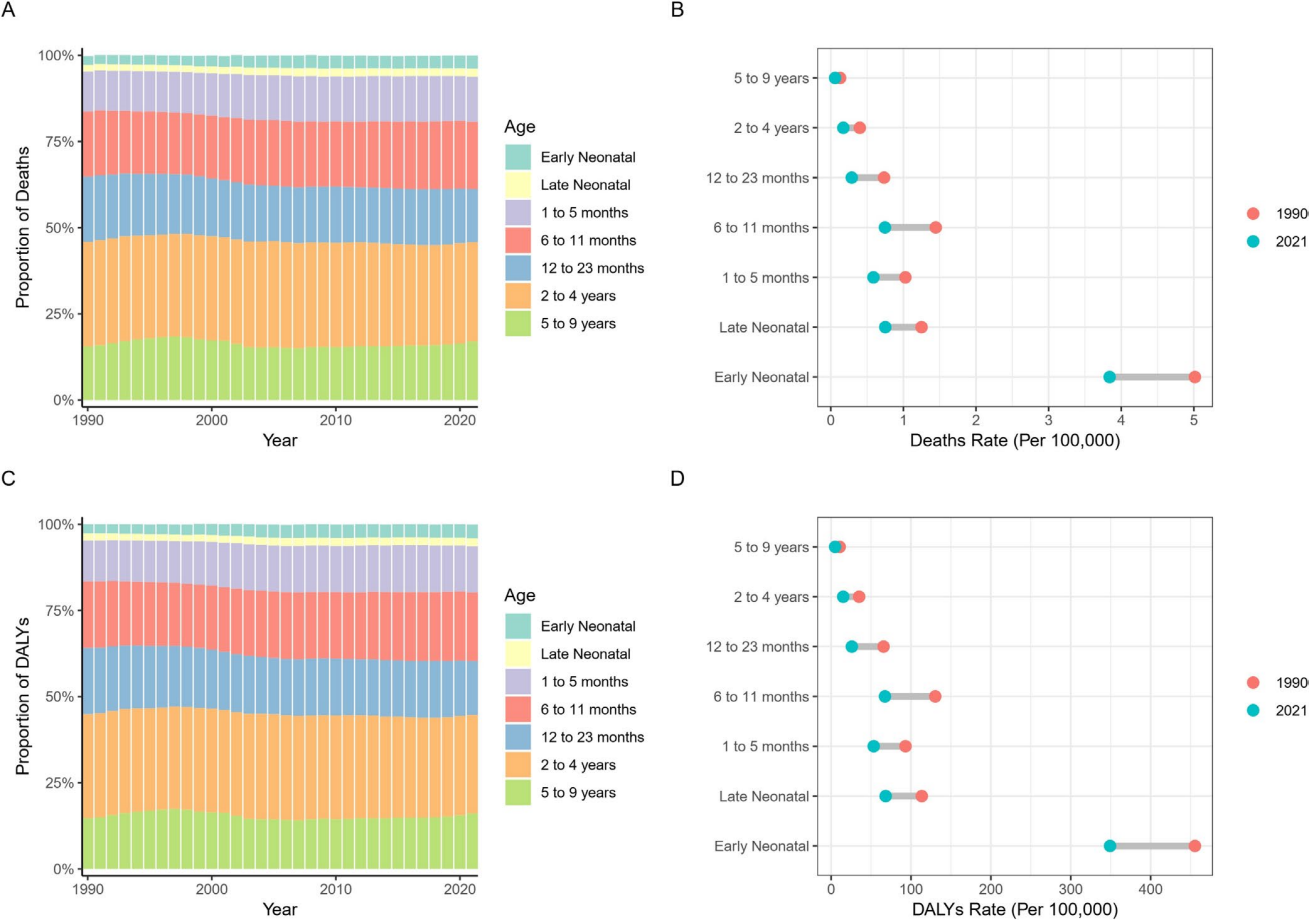
Temporal Trends in the Global Number and Rate of Hepatoblastoma‐Associated Deaths and DALYs in 1990 and 2021. Temporal changes in the number of hepatoblastoma‐associated deaths (A) and mortality rates (B) globally in all age groups from 1990 to 2021, and in the number of hepatoblastoma‐associated DALYs (C) and DALYs rates (D) globally in all age groups from 1990 to 2021.

Over the past three decades, children aged 2–4 years have consistently represented the largest proportion of global HB deaths, while the early neonatal period has accounted for the smallest proportion (Figure 2A). Additionally, from 1990 to 2021, there has been a general decline in mortality rates (per 100,000 population) across all age groups, particularly in the early neonatal group. Notably, there was a significant reduction in the percentage of deaths within the under‐5 age group; whereas the percentage of deaths in the 5–9 years age group remained largely unchanged (Figure 2B).

A similar trend is observed in the disability‐adjusted life years (DALYs) for HB. The 2–4 years age group has consistently contributed the largest proportion of DALYs globally, while the under‐5 years age group has accounted for over 70% of HB‐associated DALYs (Figure 2C). Furthermore, the rate of DALYs in the early neonatal group was the highest, with a marked decline in the DALYs rate (per 100,000 population) in the under‐5 age group from 1990 to 2021. However, the DALYs rate in the 5–9 years age group exhibited little change during this period (Figure 2D).

### 3.4 Global Trends in ASMR Versus SDI for Hepatoblastoma

**FIGURE 3:**
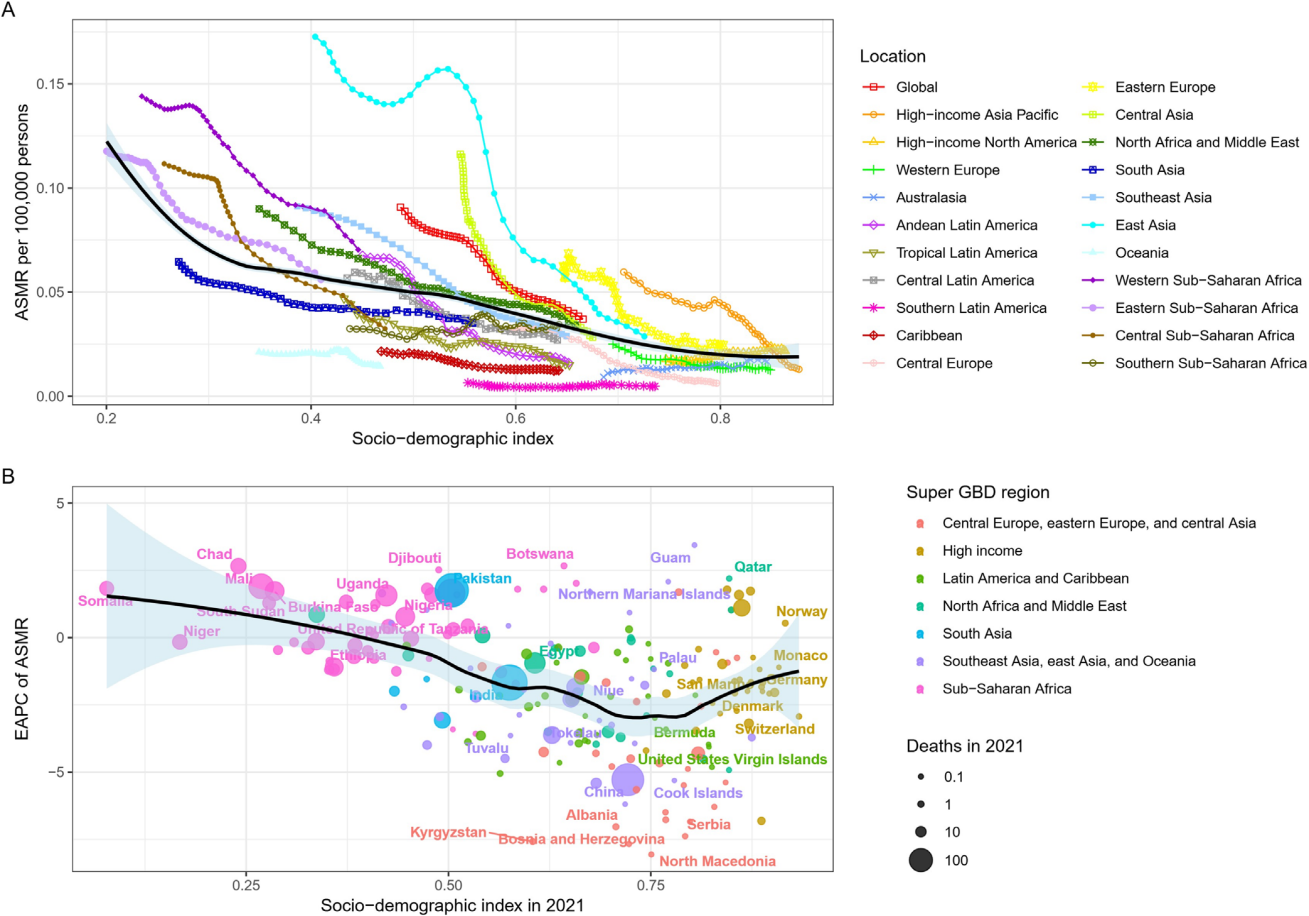
Correlation Between SDI and ASMR/EAPC for Hepatoblastoma in 2021. Correlation between ASMR and SDI for hepatoblastoma (A) and between EAPC for ASMR and SDI in 2021 (B).

The analysis revealed a significant linear relationship between the age‐standardized mortality rate (ASMR) and the socio‐demographic index (SDI) for HB. From 1990 to 2021, ASMR was negatively correlated with SDI, with higher mortality rates observed in regions with lower SDI, particularly between low and medium SDI areas. A notable upward trend in ASMR was seen as SDI decreased. In contrast, a downward trend in ASMR was observed as SDI increased from low‐medium to medium‐high SDI, with minimal change in ASMR from medium‐high to high SDI (Figure 3A).

The estimated annual percentage change (EAPC) of ASMR for HB exhibited a U‐shaped pattern with increasing SDI. Initially, the EAPC of ASMR decreased, reaching its lowest point at an SDI of approximately 0.75. Below an SDI of 0.5, the EAPC continued to decline; whereas between SDIs of 0.5 and 0.75, ASMR followed a fluctuating downward trend. In regions with an SDI greater than 0.75, a slow increase in ASMR was observed (Figure 3B).

### 3.5 Global Trends in ASDR Versus SDI for Hepatoblastoma

**FIGURE 4:**
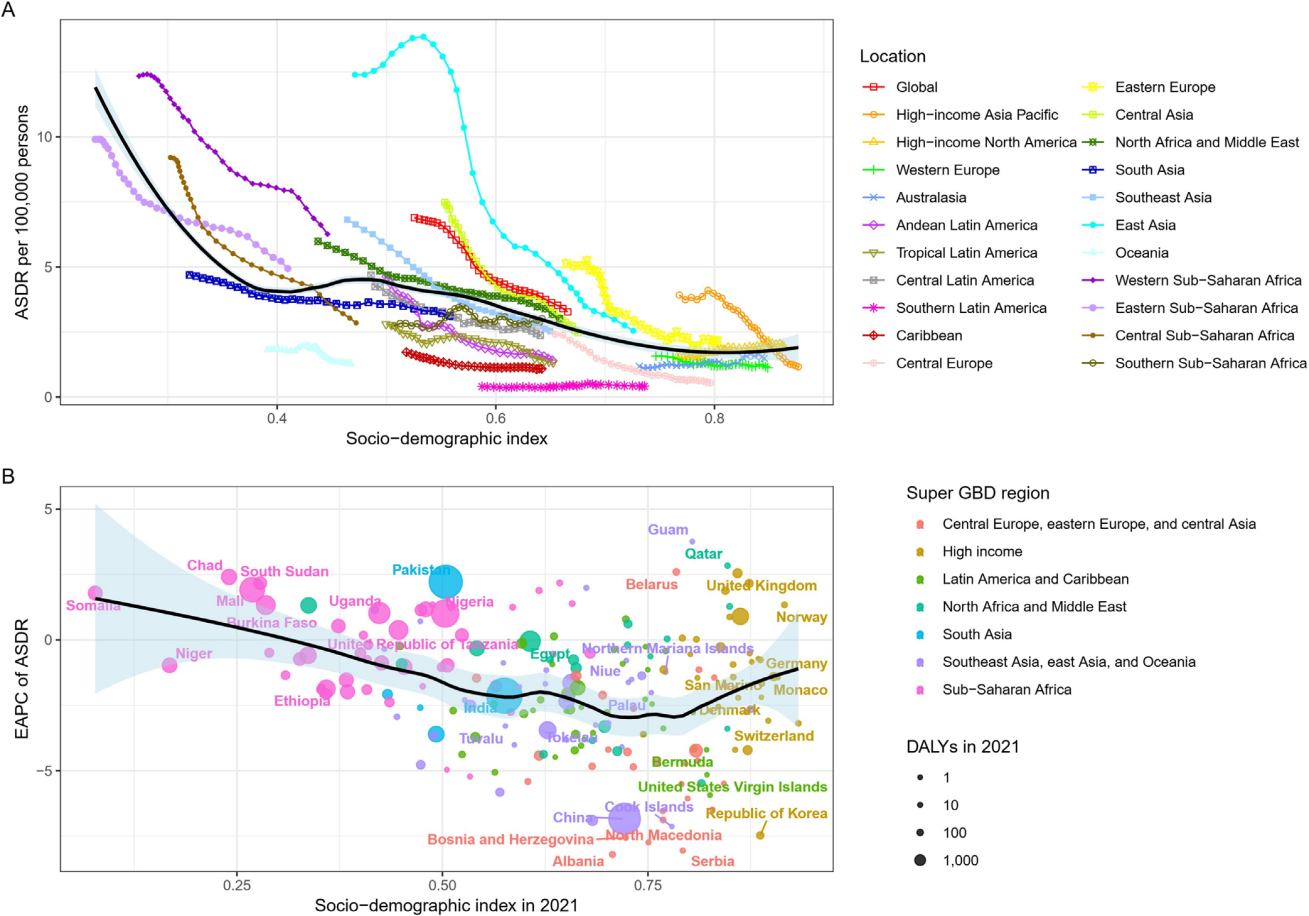
Correlation Between SDI and ASDR/EAPC for Hepatoblastoma in 2021. Correlation between ASDR and SDI for hepatoblastoma (A) and between EAPC for ASDR and SDI in 2021 (B).

The relationship between age‐standardized disability rate (ASDR) and SDI for HB demonstrated the following trends. From 1990 to 2021, ASDR was negatively correlated with SDI, with higher disability‐adjusted yearly rates in regions with low SDI. As SDI increased, ASDR for HB generally decreased, particularly in areas with an SDI below 0.4, where a significant downward trend in ASDR was observed. Conversely, when SDI was between 0.5 and 0.6, ASDR showed a fluctuating downward trend, with a gradual increase followed by a slow decrease. For SDI values greater than 0.6, a steady decline in ASDR was observed (Figure 4A).

The estimated annual percentage change (EAPC) of ASDR for HB initially decreased with increasing SDI, reaching a trough at an SDI of approximately 0.75. Below an SDI of 0.5, the EAPC of ASDR continued to decrease, while between SDIs of 0.5 and 0.75, the EAPC fluctuated downward. For SDI values above 0.75, a gradual increase in the EAPC was observed (Figure 4B).

### 3.6 Long‐Term Trends in Hepatoblastoma Worldwide, 1990–2021

**FIGURE 5:**
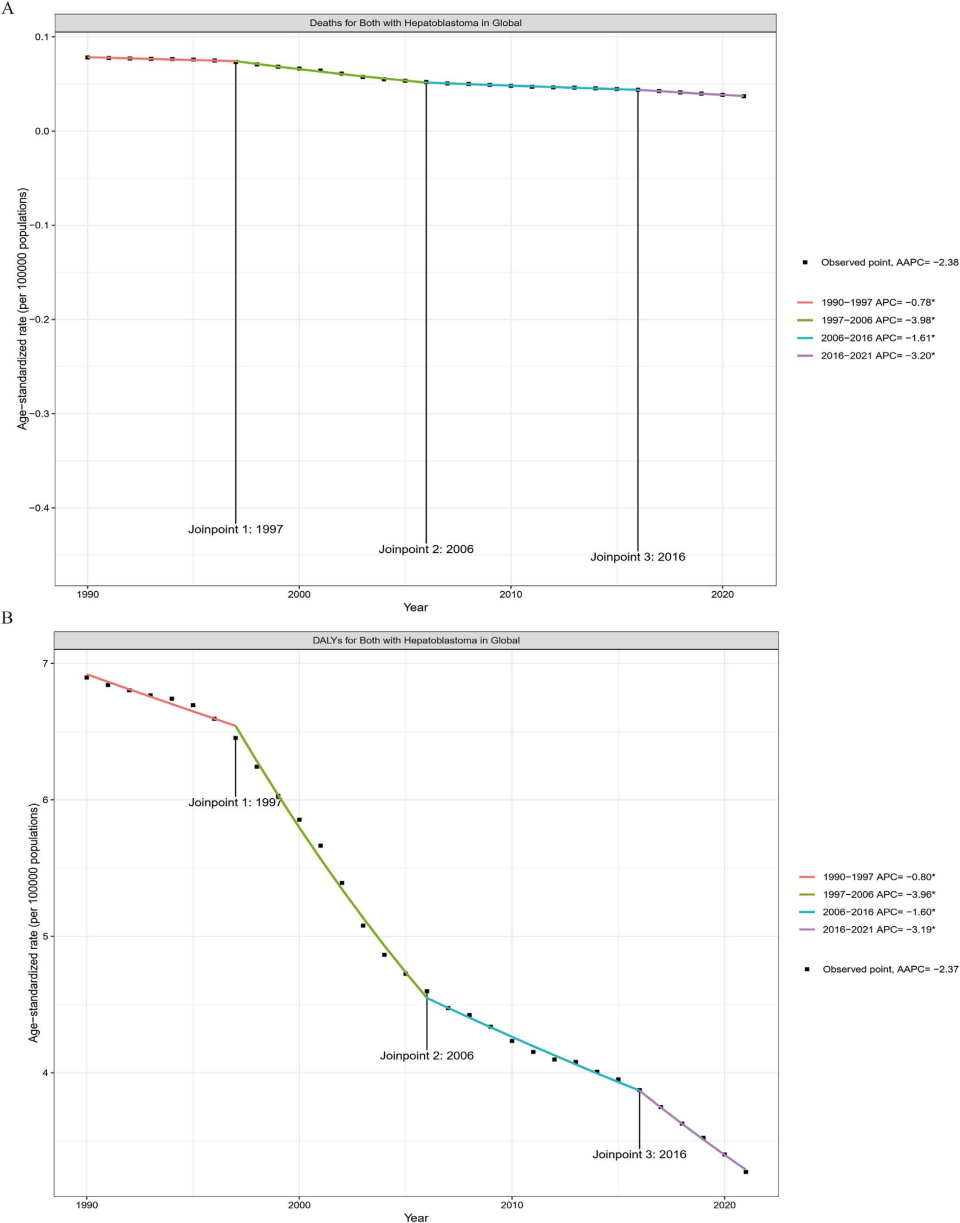
Joinpoint Regression Analysis of Global Trends in Hepatoblastoma‐Associated ASMR and ASDR (1990–2021). Joinpoint regression analysis of global age‐standardized mortality (A) and age‐standardized DALY rates (B) for hepatoblastoma from 1990 to 2021.

Over the past 32 years, the age‐standardized mortality rate (ASMR) for HB has demonstrated a consistent downward trend. From 1990 to 2021, HB‐related mortality exhibited four distinct phases. The most significant decline occurred between 1997 and 2006, with a reduction of 3.98%. In contrast, the slowest decline was observed between 1990 and 1997, with a decrease of only 0.78%. Overall, the average annual percentage change (AAPC) over the 32 years was −2.38% (Figure 5A).

A similar trend was observed in the age‐standardized disability rate (ASDR) due to HB, which also declined in four stages during this period. The most pronounced decrease occurred between 1997 and 2006, with a decline of 3.06%; while the slowest reduction was observed between 1990 and 1997, with a decline of only 0.80%. The total AAPC value for ASDR over the 32 years was −2.37% (Figure 5B).

### 3.7 Prediction of Hepatoblastoma Burden by the BAPC Model

**FIGURE 6:**
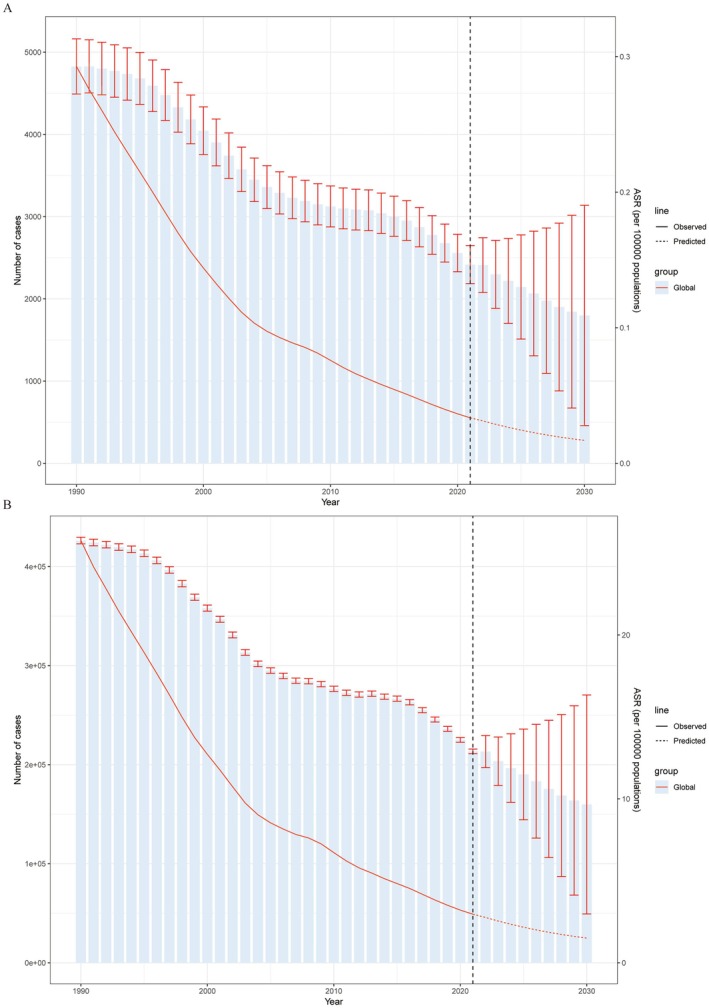
Projected Global Trends in Hepatoblastoma‐Related ASMR and ASDR, 2021–2030. Trends in global age‐standardized mortality (A) and age‐standardized DALY rates (B) for hepatoblastoma (per 100,000 population) from 2021 to 2030 based on the BAPC model: Observed (solid line) and projected (dashed line) rates. Global refers to the pooled estimates across all 204 countries and territories included in the analysis, representing the worldwide average rates.

Overall, the age‐standardized mortality rate (ASMR) and age‐standardized disability rate (ASDR) due to hepatoblastoma (HB) are projected to decrease between 2021 and 2030 (Figure 6A,B). Specifically, by 2030, the ASMR for HB is expected to decline from 0.034 (95% UI: 0.030–0.037) per 100,000 people in 2021 to 0.017 (95% UI: 0.004–0.030) per 100,000 people in 2030. Similarly, the age‐standardized disability rate (ASDR) is projected to decrease from 2.96 (95% UI: 2.93–12.73) per 100,000 people in 2021 to 1.51 (95% UI: 0.46–2.55) per 100,000 people in 2030 (Table S5).

The absolute number of deaths due to HB is expected to decrease from 2416.78 (95% UI: 2185.27–2648.30) in 2021 to 1798.14 (95% UI: 458.64–3137.63) in 2030. Similarly, the total number of disability‐adjusted life years (DALYs) is projected to decrease from 213,442.35 (95% UI: 211,069.56–215,815.14) to 159,827.22 (95% UI: 49,265.58–270,388.86) in 2030 (Table S6). In summary, the number of deaths and DALYs attributable to HB is expected to decline over the next decade.

## 4 Discussion

Hepatoblastoma (HB) is the most common primary malignant liver tumor in children, occurring predominantly in children under 5 years of age [20]. It is typically caused by abnormalities in the development of undifferentiated hepatocytes and may be associated with genetic susceptibility. Certain inherited disorders, such as Fanconi anemia and Beckwith‐Wiedemann syndrome, increase the risk of developing HB [21]. In addition, environmental factors during pregnancy, low birth weight, and some viral hepatitis infections have been identified as possible associations with the development of HB [22]. If diagnosed and treated early, HB has a favorable prognosis, with a 5‐year survival rate of 70%–80% [23]. However, in the absence of early clinical symptoms, diagnosis often occurs at an advanced stage, particularly in patients with distant metastases, resulting in a lower survival rate [24]. While children with advanced HB have a cure rate of 50%–70%, those with advanced hepatocellular carcinoma (HCC) have a 5‐year survival rate of less than 20% [25]. Treatment options for HB include surgical resection, chemotherapy, and liver transplantation [26]. Although many cases can be effectively treated with surgery and chemotherapy, some children still face recurrence or drug resistance during treatment [27]. Early diagnosis, precise treatment, and long‐term follow‐up are essential for improving outcomes.

Despite the relatively low incidence of HB, its treatment is complex and costly, particularly when liver transplantation is required, placing significant financial pressure on both the patient's family and society. Additionally, long‐term rehabilitation and monitoring increase the burden on families [28]. Therefore, a comprehensive understanding of the global burden of HB is essential for developing appropriate public health strategies.

While Guo et al. recently analyzed hepatoblastoma epidemiology from 1990 to 2021 using GBD 2021 data, our study provides several distinct contributions [29]. First, we extend the analysis through future projections to 2030 using Bayesian age‐period‐cohort modeling, offering valuable insights for health planning and resource allocation. Second, our study specifically examines regional disparities in relation to socio‐demographic index (SDI) levels, providing a more detailed analysis of how socioeconomic factors influence hepatoblastoma burden across different regions. Third, we incorporate a comprehensive analysis of the relationship between healthcare improvements and observed mortality trends, including specific interventions that may have contributed to improved outcomes. These additional analytical dimensions complement the existing literature and provide enhanced guidance for targeted prevention and management strategies.

Primary liver cancers include hepatitis B, hepatitis C, alcohol, NASH, hepatoblastoma, and other causes [30]. Previous studies have examined the global burden of hepatitis B, hepatitis C, alcohol, NASH‐induced liver cancer, and total liver cancer. Tomi Akinyemiju et al. reported on the global burden of primary liver cancer and its association with hepatitis B, hepatitis C, and alcohol [6]. Additionally, Pojsakorn et al. analyzed the global burden of NASH‐associated hepatocellular carcinoma [9]. However, the global disease burden and future trends of hepatoblastoma remain understudied. This study differs from previous research by being the first to utilize Joinpoint regression and Bayesian age‐period‐cohort (BAPC) modeling, based on the Global Burden of Disease (GBD) 2021 data, to analyze global hepatoblastoma mortality trends. We also discuss variations in these trends by gender, age, and region.

Our study revealed that, in 2021, the global number of HB‐related deaths was 2416.17, representing a 49.96% decrease from 1990. During the same period, the mortality rate for all age groups decreased by 66.18%, and the age‐standardized mortality rate (ASMR) decreased by 52.63%. Furthermore, the net drift in mortality showed a decreasing trend, likely reflecting advancements in medical technology and improvements in lifestyle [31].

We also explored regional differences in the burden of hepatoblastoma and analyzed the relationship between the age‐standardized rate of hepatoblastoma and the Socio‐Demographic Index (SDI). Notably, regions with higher SDI exhibited lower ASMR, suggesting that better healthcare infrastructure, higher public health awareness, and greater healthcare investment may contribute to improved outcomes. In contrast, countries with low SDI would benefit from strengthening basic health infrastructure, promoting healthy lifestyles, and improving access to early screening and intervention. In regions with higher SDI, the focus should be on strengthening health systems, promoting standardized disease management programs, and ensuring rational treatment protocols.

The differences in hepatoblastoma burden across SDI levels highlight major global inequalities in pediatric cancer care. In low‐SDI regions, higher mortality and DALY rates are largely due to delayed diagnosis, limited access to pediatric oncology services, and a lack of curative treatments such as surgery or liver transplantation. Early symptoms are often misdiagnosed as common childhood illnesses, leading to late‐stage presentation. Structural healthcare barriers—including shortages of personnel, diagnostic tools, and essential medicines—are compounded by poverty, malnutrition, and low health literacy, which reduce treatment adherence. In contrast, high‐SDI regions benefit from early detection, standardized therapies, and comprehensive care, resulting in better outcomes. These disparities call for targeted global efforts to strengthen diagnostic capacity, expand access to treatment, and implement context‐appropriate early detection strategies in low‐resource settings.

Globally, males exhibited slightly higher deaths and DALYs from hepatoblastoma, consistent with the well‐documented male predominance in incidence. This disparity may be linked to biological and developmental factors. Androgens such as testosterone may promote hepatocyte proliferation or increase vulnerability to oncogenic stimuli during early liver development. Additionally, males are more likely to be born preterm or with very low birth weight—both known risk factors for hepatoblastoma. Sex‐based differences in immune maturation and liver metabolism may also contribute, though mechanisms remain unclear.

This male predominance is most pronounced in high‐SDI regions (male‐to‐female ratio ~1.5:1), where better diagnostic and treatment access may reveal the underlying biological trends more accurately. However, in low‐SDI regions, this pattern reverses, with females bearing a relatively higher disease burden. This is unlikely due to biological susceptibility but may reflect sociocultural and systemic inequities. In many low‐resource settings, healthcare prioritization may favor boys, leading to delayed diagnosis or early treatment discontinuation in girls. Economic hardship and low health literacy may further limit treatment adherence among female patients. These findings highlight the importance of gender‐sensitive public health strategies that promote equitable access to timely diagnosis and treatment, particularly in settings with constrained resources.

Several specific interventions and policies may have contributed to the observed improvements in hepatoblastoma outcomes globally. First, advances in surgical techniques, including improved liver resection methods and the increased availability of liver transplantation programs, have significantly enhanced treatment outcomes for hepatoblastoma patients. Many high‐income countries have established specialized pediatric liver transplant centers with standardized protocols. Second, the development and implementation of standardized chemotherapy regimens, such as those recommended by international collaborative groups like the International Childhood Liver Tumor Strategy Group (SIOPEL) and the Children's Oncology Group (COG), have improved survival rates. Third, enhanced early detection strategies, including improved awareness among healthcare providers about hepatoblastoma presentations and the use of alpha‐fetoprotein (AFP) screening in high‐risk populations, have facilitated earlier diagnosis and treatment initiation. Fourth, the establishment of multidisciplinary care teams in many healthcare systems has optimized treatment planning and coordination. Finally, improvements in supportive care, including better management of chemotherapy‐related toxicities and enhanced nutritional support, have contributed to improved patient outcomes.

Based on our findings, specific recommendations for clinical management and policy formulation include: establishing regional pediatric oncology centers with hepatoblastoma expertise and telemedicine consultation capabilities in low SDI regions; implementing standardized AFP screening protocols for high‐risk populations, particularly in regions with high hepatoblastoma prevalence; developing risk‐stratified treatment algorithms that account for regional resource availability; establishing international treatment consortiums to facilitate knowledge transfer; and investing in healthcare provider training programs focused on early recognition and management of pediatric liver tumors in resource‐limited settings.

We examined temporal trends in the HB burden to assess the effectiveness of national health interventions. A downward trend in HB‐related mortality was observed, indicating that the global burden of HB improved over the study period. Additionally, we found that the number of deaths and disability‐adjusted life years (DALYs) was higher in males than in females. This higher male mortality may be linked to the effects of sex hormones and genetic factors [32]. Androgens may stimulate hepatocyte proliferation, increasing the risk of hepatoblastoma, while estrogens have tumor‐suppressing effects [33]. Genetic disorders like Beckwith‐Wiedemann syndrome, more common in males, may also contribute [21]. Gender differences could reflect in healthcare access [34]. The overall decline in HB mortality likely reflects advancements in understanding, treatment, and prevention.

This study has some limitations. First, it specifically analyzed HB as a single risk factor, without considering the influence of multiple coexisting risk factors on disease outcomes. Second, due to the lack of staging and grading data in the GBD database, we were unable to assess the mortality and prognosis of HB based on different stages. This limitation may hinder timely and effective medical decision‐making. Third, this study relies entirely on modeled estimates and secondary data from the GBD 2021 database, which may be subject to reporting biases and data quality issues. The accuracy and completeness of national cancer registry data vary significantly across countries, particularly in low‐ and middle‐income regions where hepatoblastoma surveillance systems may be less developed. This heterogeneity in data quality could potentially lead to underestimation or overestimation of the true disease burden in certain regions, especially in low SDI areas. Additionally, the GBD methodology involves complex modeling techniques to address data gaps, which may introduce uncertainty in the estimates for rare diseases like hepatoblastoma. Lastly, data collection difficulties and quality issues in low‐income or sparsely populated countries may have affected the accuracy of projections from the Bayesian age‐period‐cohort (BAPC) model through 2030. Improved epidemiological data quality in these regions is essential for better modeling [11, 35].

Future research should focus on investigating genetic and environmental risk factors specific to high‐burden regions to inform prevention strategies, developing cost‐effective screening and treatment protocols adapted for middle and low SDI settings, conducting longitudinal studies to evaluate intervention effectiveness, exploring novel therapeutic approaches implementable in resource‐constrained environments, and establishing robust cancer registration systems in underserved regions to improve epidemiological monitoring.

## 5 Conclusions

Our study highlights the increasing burden of HB in regions like West Africa, despite the global decrease in HB burden from 1990 to 2021. Notably, children aged 5 and under account for a significant and growing proportion of HB‐related deaths. Additionally, both global HB‐related deaths and DALYs are lower for females than for males. The burden of HB has decreased more significantly in regions with high SDI compared to those with medium or low SDI. These findings underscore the need for targeted intervention strategies tailored to different regional and socio‐economic contexts.

## Author Contributions

F.W. and X.Y. primarily contributed to the research ideas and revisions of the manuscript. X.Y. and Z.Z. were mainly involved in manuscript writing and data processing. Q.W. and S.K. primarily contributed to literature retrieval and data processing. All authors agreed on the final version of the manuscript.

## Ethics Statement

This study does not require ethical approval, as it does not involve human participants, personal data, or animal subjects. All data utilized in this research are publicly available and de‐identified, thus exempting them from the need for ethical review.

## Consent

The authors have nothing to report.

## Conflicts of Interest

The authors declare no Conflicts of Interest.

## Supporting information


**Data S1:** cam471163‐sup‐0001‐DataS1.docx.

## Data Availability

The data that support the findings of this study are available from the corresponding author upon reasonable request.
