# Supplementary material for: Global Burden of Hepatoblastoma From 1990 to 2021 and Projection to 2030
Source: Cancer Med. 2025 Aug 19;14(16):e71163. doi: 10.1002/cam4.71163 (PMC12361928; doi:10.1002/cam4.71163)
Supplement: Supplementary file 1 — Data S1: cam471163‐sup‐0001‐DataS1.docx. [file CAM4-14-e71163-s001.docx]

**Supplementary Material**

***Global burden of hepatoblastoma from 1990 to 2021 and projection to 2030***

**Content of Supplemental Material**

**Supplementary Tables**

- **Supplementary Table S1.** Age-standardized mortality rates of Hepatoblastoma by country and region in 1990.
- **Supplementary Table S2.** Age-standardized mortality rates of Hepatoblastoma by country and region in 2021.
- **Supplementary Table S3.** Age-standardized disability rates of Hepatoblastoma by country and region in 1990.
- **Supplementary Table S4.** Age-standardized disability rates of Hepatoblastoma by country and region in 1990.
- **Supplementary Table S5.** Prediction of hepatoblastoma age-standardized rates by sex, 2021-2030
- **Supplementary Table S6.** Prediction of hepatoblastoma numbers, 2021-2030

**Supplementary Table S1**: Age-standardized mortality rates of Hepatoblastoma by country and region in 1990

| location | val | upper | lower | val2 |
| --- | --- | --- | --- | --- |
| Tokelau | 0.009350931 | 0.02113496 | 0.004166128 | <0.01 |
| France | 0.021445836 | 0.024313639 | 0.018878925 | 0.02 to <0.02 |
| Eritrea | 0.060911658 | 0.107840834 | 0.031468399 | 0.05 to <0.08 |
| Honduras | 0.040075591 | 0.058048501 | 0.027380843 | 0.04 to <0.05 |
| Mexico | 0.051675129 | 0.058261587 | 0.046615656 | 0.04 to <0.05 |
| Bhutan | 0.076239259 | 0.141717656 | 0.031489729 | 0.08 to <0.13 |
| Germany | 0.015212087 | 0.018329729 | 0.012820603 | 0.01 to <0.02 |
| Ethiopia | 0.080511787 | 0.133020785 | 0.040129799 | 0.08 to <0.13 |
| Argentina | 0.004695846 | 0.005494193 | 0.003978392 | <0.01 |
| Myanmar | 0.053702709 | 0.113501202 | 0.009603438 | 0.05 to <0.08 |
| India | 0.045834088 | 0.061884845 | 0.028759298 | 0.04 to <0.05 |
| Eswatini | 0.029584491 | 0.048029033 | 0.015506174 | 0.02 to <0.04 |
| Guyana | 0.016250749 | 0.020192735 | 0.012818193 | 0.01 to <0.02 |
| Greece | 0.019295817 | 0.021032386 | 0.017778613 | 0.02 to <0.02 |
| South Africa | 0.02372103 | 0.031963937 | 0.015535941 | 0.02 to <0.04 |
| North Macedonia | 0.097861197 | 0.125295776 | 0.067847378 | 0.08 to <0.13 |
| Mauritania | 0.175856357 | 0.398939089 | 0.047045272 | 0.13 to <0.88 |
| Philippines | 0.094192513 | 0.122969724 | 0.06342546 | 0.08 to <0.13 |
| Kenya | 0.056680918 | 0.078285325 | 0.034018586 | 0.05 to <0.08 |
| Hungary | 0.014074559 | 0.017257672 | 0.011493593 | 0.01 to <0.02 |
| Nicaragua | 0.083107577 | 0.108542216 | 0.063559561 | 0.08 to <0.13 |
| Japan | 0.028957826 | 0.030367549 | 0.027863309 | 0.02 to <0.04 |
| Madagascar | 0.070817935 | 0.114519846 | 0.042939432 | 0.05 to <0.08 |
| Lebanon | 0.027376727 | 0.040843957 | 0.017585528 | 0.02 to <0.04 |
| Nepal | 0.049393278 | 0.081415473 | 0.017226588 | 0.04 to <0.05 |
| Tuvalu | 0.020164362 | 0.035784405 | 0.010352623 | 0.02 to <0.02 |
| Haiti | 0.029739908 | 0.059485602 | 0.010952444 | 0.02 to <0.04 |
| Uruguay | 0.00348741 | 0.004343766 | 0.002815775 | <0.01 |
| Bermuda | 0.00893891 | 0.011456227 | 0.006705988 | <0.01 |
| Panama | 0.043040147 | 0.050309148 | 0.035494951 | 0.04 to <0.05 |
| Republic of Korea | 0.075825068 | 0.110157841 | 0.04564487 | 0.05 to <0.08 |
| Mozambique | 0.10061778 | 0.154073204 | 0.055162398 | 0.08 to <0.13 |
| Brazil | 0.031606019 | 0.037343829 | 0.027240693 | 0.02 to <0.04 |
| Italy | 0.026404385 | 0.027940642 | 0.025019169 | 0.02 to <0.04 |
| Saint Vincent and the Grenadines | 0.020009088 | 0.025231675 | 0.015718377 | 0.02 to <0.02 |
| Rwanda | 0.10400902 | 0.167261793 | 0.053212247 | 0.08 to <0.13 |
| Cameroon | 0.119842058 | 0.192423754 | 0.060697077 | 0.08 to <0.13 |
| Armenia | 0.047491345 | 0.06010669 | 0.038178731 | 0.04 to <0.05 |
| Angola | 0.242498321 | 0.465832069 | 0.086057502 | 0.13 to <0.88 |
| United States Virgin Islands | 0.006016158 | 0.008840822 | 0.003858913 | <0.01 |
| Luxembourg | 0.015994602 | 0.017950559 | 0.014139335 | 0.01 to <0.02 |
| New Zealand | 0.017803846 | 0.021546222 | 0.014690285 | 0.02 to <0.02 |
| Uganda | 0.183481078 | 0.266008427 | 0.118082355 | 0.13 to <0.88 |
| Central African Republic | 0.138201391 | 0.225551627 | 0.073159003 | 0.13 to <0.88 |
| Norway | 0.013344984 | 0.014393209 | 0.012286004 | 0.01 to <0.02 |
| Niger | 0.217326524 | 0.344124787 | 0.129324128 | 0.13 to <0.88 |
| Montenegro | 0.036043222 | 0.05027873 | 0.024170845 | 0.02 to <0.04 |
| Paraguay | 0.036924644 | 0.054054686 | 0.024947912 | 0.04 to <0.05 |
| Morocco | 0.009559803 | 0.014766338 | 0.006020287 | <0.01 |
| Gambia | 0.361052095 | 0.53266836 | 0.239741589 | 0.13 to <0.88 |
| American Samoa | 0.005799705 | 0.010459832 | 0.003276719 | <0.01 |
| Ecuador | 0.05112661 | 0.062030905 | 0.042314269 | 0.04 to <0.05 |
| Belgium | 0.013316844 | 0.015581083 | 0.011327285 | 0.01 to <0.02 |
| Bahrain | 0.023739574 | 0.032437265 | 0.017376393 | 0.02 to <0.04 |
| Brunei Darussalam | 0.025574706 | 0.037665329 | 0.017159078 | 0.02 to <0.04 |
| Namibia | 0.026242888 | 0.0410875 | 0.014626709 | 0.02 to <0.04 |
| Equatorial Guinea | 0.042676285 | 0.090800508 | 0.016105258 | 0.04 to <0.05 |
| Iceland | 0.009165587 | 0.010872478 | 0.007658149 | <0.01 |
| Jamaica | 0.011296455 | 0.014653726 | 0.008205562 | 0.01 to <0.02 |
| Portugal | 0.023126627 | 0.027247629 | 0.018940025 | 0.02 to <0.04 |
| Gabon | 0.073186497 | 0.129517549 | 0.038365798 | 0.05 to <0.08 |
| United Kingdom | 0.011988372 | 0.012472241 | 0.01151263 | 0.01 to <0.02 |
| Zambia | 0.202192688 | 0.289273286 | 0.134420795 | 0.13 to <0.88 |
| Spain | 0.024272705 | 0.027858525 | 0.021197875 | 0.02 to <0.04 |
| Chile | 0.004524546 | 0.005924448 | 0.003546108 | <0.01 |
| Mali | 0.395836308 | 0.604956676 | 0.174451862 | 0.13 to <0.88 |
| El Salvador | 0.032991755 | 0.041440204 | 0.025412061 | 0.02 to <0.04 |
| Jordan | 0.017711114 | 0.029931586 | 0.010934866 | 0.02 to <0.02 |
| Egypt | 0.131748383 | 0.189496233 | 0.080763076 | 0.13 to <0.88 |
| Belize | 0.025712821 | 0.031805472 | 0.021352267 | 0.02 to <0.04 |
| Cyprus | 0.013738805 | 0.021601884 | 0.008598285 | 0.01 to <0.02 |
| Libya | 0.065607178 | 0.093360832 | 0.043943937 | 0.05 to <0.08 |
| Djibouti | 0.070580323 | 0.130351801 | 0.040633068 | 0.05 to <0.08 |
| Denmark | 0.008841453 | 0.010068952 | 0.00780579 | <0.01 |
| Nigeria | 0.069488673 | 0.094733352 | 0.043400229 | 0.05 to <0.08 |
| Palestine | 0.049257594 | 0.072612547 | 0.031063875 | 0.04 to <0.05 |
| Saint Lucia | 0.010794035 | 0.013569218 | 0.008282625 | 0.01 to <0.02 |
| Azerbaijan | 0.064361935 | 0.112549103 | 0.033173057 | 0.05 to <0.08 |
| Pakistan | 0.085404189 | 0.125983466 | 0.049104028 | 0.08 to <0.13 |
| Kuwait | 0.038382856 | 0.047306419 | 0.031386194 | 0.04 to <0.05 |
| Canada | 0.012227501 | 0.013657403 | 0.010907828 | 0.01 to <0.02 |
| Malawi | 0.251556634 | 0.3796385 | 0.156923756 | 0.13 to <0.88 |
| Thailand | 0.126080807 | 0.169533272 | 0.092234914 | 0.13 to <0.88 |
| Ireland | 0.00867384 | 0.010093126 | 0.007464195 | <0.01 |
| Cook Islands | 0.016424309 | 0.028090667 | 0.00943968 | 0.02 to <0.02 |
| Venezuela (Bolivarian Republic of) | 0.036555475 | 0.039116276 | 0.033916043 | 0.02 to <0.04 |
| China | 0.142616546 | 0.174772829 | 0.118542657 | 0.13 to <0.88 |
| Senegal | 0.107490108 | 0.161275025 | 0.063379394 | 0.08 to <0.13 |
| Kazakhstan | 0.059608299 | 0.067929466 | 0.052245471 | 0.05 to <0.08 |
| Viet Nam | 0.165512194 | 0.236322668 | 0.109451408 | 0.13 to <0.88 |
| Greenland | 0.052798247 | 0.074266177 | 0.037829312 | 0.05 to <0.08 |
| Timor-Leste | 0.056623425 | 0.10195745 | 0.012483352 | 0.05 to <0.08 |
| Mauritius | 0.01498862 | 0.016638322 | 0.013578948 | 0.01 to <0.02 |
| Sierra Leone | 0.248480394 | 0.376127632 | 0.13007479 | 0.13 to <0.88 |
| Benin | 0.201689167 | 0.314746755 | 0.126758494 | 0.13 to <0.88 |
| Romania | 0.037536743 | 0.047567757 | 0.029878263 | 0.04 to <0.05 |
| Slovakia | 0.022145291 | 0.033435212 | 0.014343779 | 0.02 to <0.02 |
| Oman | 0.021994034 | 0.035018911 | 0.012910672 | 0.02 to <0.02 |
| Serbia | 0.037801928 | 0.064238775 | 0.01932569 | 0.04 to <0.05 |
| Saudi Arabia | 0.052512644 | 0.09195174 | 0.032260339 | 0.05 to <0.08 |
| Democratic People's Republic of Korea | 0.102755734 | 0.205235474 | 0.048836565 | 0.08 to <0.13 |
| Guam | 0.005455604 | 0.007594718 | 0.004059333 | <0.01 |
| Togo | 0.061713643 | 0.093056853 | 0.038080923 | 0.05 to <0.08 |
| Zimbabwe | 0.055774301 | 0.092197103 | 0.029886756 | 0.05 to <0.08 |
| Malta | 0.007988439 | 0.009547566 | 0.006258917 | <0.01 |
| Northern Mariana Islands | 0.00349676 | 0.006125529 | 0.001925955 | <0.01 |
| Sao Tome and Principe | 0.055894121 | 0.083363431 | 0.035404949 | 0.05 to <0.08 |
| Georgia | 0.024389776 | 0.028866191 | 0.020081553 | 0.02 to <0.04 |
| Sri Lanka | 0.007598828 | 0.010124114 | 0.005533148 | <0.01 |
| United States of America | 0.017860685 | 0.018276098 | 0.017457761 | 0.02 to <0.02 |
| Qatar | 0.029758029 | 0.042723146 | 0.019895406 | 0.02 to <0.04 |
| South Sudan | 0.122744541 | 0.209292204 | 0.049191676 | 0.08 to <0.13 |
| Israel | 0.013050534 | 0.01508702 | 0.011172906 | 0.01 to <0.02 |
| Bolivia (Plurinational State of) | 0.081172327 | 0.128977253 | 0.04035139 | 0.08 to <0.13 |
| Bahamas | 0.014742705 | 0.018042176 | 0.011915038 | 0.01 to <0.02 |
| Chad | 0.12744334 | 0.205225707 | 0.076013346 | 0.13 to <0.88 |
| Tajikistan | 0.061860576 | 0.101712642 | 0.026117182 | 0.05 to <0.08 |
| Taiwan (Province of China) | 0.03900492 | 0.044253749 | 0.034514254 | 0.04 to <0.05 |
| Tunisia | 0.038095446 | 0.054210672 | 0.024171547 | 0.04 to <0.05 |
| Burkina Faso | 0.219938394 | 0.367995035 | 0.131057761 | 0.13 to <0.88 |
| Fiji | 0.008331972 | 0.016531659 | 0.004460361 | <0.01 |
| Kyrgyzstan | 0.093444088 | 0.126658036 | 0.068257293 | 0.08 to <0.13 |
| Seychelles | 0.01545889 | 0.023865691 | 0.010561161 | 0.01 to <0.02 |
| Suriname | 0.017573844 | 0.025057097 | 0.011638436 | 0.02 to <0.02 |
| Kiribati | 0.035310631 | 0.061006296 | 0.019677183 | 0.02 to <0.04 |
| Indonesia | 0.0369826 | 0.062620499 | 0.012183603 | 0.04 to <0.05 |
| Nauru | 0.022978788 | 0.038544777 | 0.013803627 | 0.02 to <0.04 |
| Poland | 0.004342826 | 0.004867977 | 0.003928687 | <0.01 |
| Singapore | 0.031085376 | 0.037441376 | 0.025791552 | 0.02 to <0.04 |
| Mongolia | 0.884053448 | 1.372720307 | 0.53657893 | 0.13 to <0.88 |
| Monaco | 0.025046252 | 0.04216112 | 0.01458175 | 0.02 to <0.04 |
| Latvia | 0.017387094 | 0.02033325 | 0.014753045 | 0.02 to <0.02 |
| Australia | 0.012361118 | 0.01391087 | 0.010861463 | 0.01 to <0.02 |
| Turkey | 0.033923863 | 0.050361971 | 0.023604681 | 0.02 to <0.04 |
| Sweden | 0.016496004 | 0.019046953 | 0.014291258 | 0.02 to <0.02 |
| Cabo Verde | 0.063870211 | 0.099001899 | 0.029584881 | 0.05 to <0.08 |
| Lao People's Democratic Republic | 0.115817735 | 0.199695573 | 0.035188063 | 0.08 to <0.13 |
| Barbados | 0.009072552 | 0.011028798 | 0.007262409 | <0.01 |
| Turkmenistan | 0.089632139 | 0.102929693 | 0.075514037 | 0.08 to <0.13 |
| United Arab Emirates | 0.022368165 | 0.037073177 | 0.012629617 | 0.02 to <0.02 |
| Netherlands | 0.00770782 | 0.008640816 | 0.006851633 | <0.01 |
| Albania | 0.20911852 | 0.30060789 | 0.138798959 | 0.13 to <0.88 |
| Algeria | 0.037615844 | 0.054389825 | 0.027008911 | 0.04 to <0.05 |
| Democratic Republic of the Congo | 0.066236163 | 0.131077011 | 0.027549636 | 0.05 to <0.08 |
| Coted'Ivoire | 0.151706375 | 0.250865545 | 0.085052522 | 0.13 to <0.88 |
| Guinea-Bissau | 0.189377095 | 0.320456591 | 0.094407131 | 0.13 to <0.88 |
| Malaysia | 0.021763459 | 0.034918447 | 0.012768554 | 0.02 to <0.02 |
| Uzbekistan | 0.040838374 | 0.055936169 | 0.028791126 | 0.04 to <0.05 |
| Georgia | 0.016226668 | 0.01728007 | 0.015231954 | 0.01 to <0.02 |
| Somalia | 0.120045868 | 0.206921787 | 0.061838869 | 0.08 to <0.13 |
| Trinidad and Tobago | 0.01342104 | 0.01580246 | 0.011305179 | 0.01 to <0.02 |
| Cambodia | 0.101006792 | 0.176316686 | 0.053691754 | 0.08 to <0.13 |
| Micronesia (Federated States of) | 0.012642188 | 0.022294505 | 0.006714805 | 0.01 to <0.02 |
| Marshall Islands | 0.005167644 | 0.011767429 | 0.002217522 | <0.01 |
| United Republic of Tanzania | 0.124859388 | 0.183698556 | 0.07809309 | 0.08 to <0.13 |
| Iran (Islamic Republic of) | 0.063641972 | 0.081400026 | 0.047541604 | 0.05 to <0.08 |
| Sudan | 0.113566455 | 0.187926214 | 0.058141185 | 0.08 to <0.13 |
| Slovenia | 0.019299593 | 0.022697668 | 0.016432103 | 0.02 to <0.02 |
| Belarus | 0.013539895 | 0.017638811 | 0.01052624 | 0.01 to <0.02 |
| Puerto Rico | 0.009149102 | 0.011168159 | 0.007476135 | <0.01 |
| Samoa | 0.035111591 | 0.060298357 | 0.02042167 | 0.02 to <0.04 |
| Syrian Arab Republic | 0.061236614 | 0.082225856 | 0.039676332 | 0.05 to <0.08 |
| Andorra | 0.095158814 | 0.166056886 | 0.040310365 | 0.08 to <0.13 |
| Palau | 0.017485139 | 0.035291 | 0.007868908 | 0.02 to <0.02 |
| Niue | 0.015335723 | 0.030087946 | 0.007254136 | 0.01 to <0.02 |
| Ukraine | 0.04480604 | 0.057124618 | 0.035481497 | 0.04 to <0.05 |
| Austria | 0.016665977 | 0.019334227 | 0.014336978 | 0.02 to <0.02 |
| San Marino | 0.017246787 | 0.02639371 | 0.011033892 | 0.02 to <0.02 |
| Congo | 0.12019582 | 0.209419617 | 0.061235941 | 0.08 to <0.13 |
| Colombia | 0.036587129 | 0.043445601 | 0.030854812 | 0.04 to <0.05 |
| Afghanistan | 0.140985522 | 0.253193211 | 0.047028628 | 0.13 to <0.88 |
| Dominican Republic | 0.025483979 | 0.03806634 | 0.013916906 | 0.02 to <0.04 |
| Bulgaria | 0.044736701 | 0.057700728 | 0.03397593 | 0.04 to <0.05 |
| Bangladesh | 0.067463764 | 0.11309868 | 0.02528466 | 0.05 to <0.08 |
| Bosnia and Herzegovina | 0.027495638 | 0.037340869 | 0.019661863 | 0.02 to <0.04 |
| Dominica | 0.008497127 | 0.013066779 | 0.005390393 | <0.01 |
| Antigua and Barbuda | 0.00886782 | 0.011106344 | 0.00695527 | <0.01 |
| Switzerland | 0.021915145 | 0.025258597 | 0.018759175 | 0.02 to <0.02 |
| Iraq | 0.055686709 | 0.077846175 | 0.036741718 | 0.05 to <0.08 |
| Comoros | 0.083331037 | 0.133302676 | 0.044179591 | 0.08 to <0.13 |
| Liberia | 0.26171829 | 0.439468903 | 0.099280914 | 0.13 to <0.88 |
| Croatia | 0.014391025 | 0.018952065 | 0.010559919 | 0.01 to <0.02 |
| Maldives | 0.036991092 | 0.063918239 | 0.012906363 | 0.04 to <0.05 |
| Solomon Islands | 0.015184773 | 0.035680285 | 0.0056589 | 0.01 to <0.02 |
| Tonga | 0.050660932 | 0.099482819 | 0.026552626 | 0.04 to <0.05 |
| Estonia | 0.021173721 | 0.025687301 | 0.017056907 | 0.02 to <0.02 |
| Peru | 0.043452918 | 0.063647826 | 0.026434571 | 0.04 to <0.05 |
| Republic of Moldova | 0.147233047 | 0.171347209 | 0.127134268 | 0.13 to <0.88 |
| Botswana | 0.014787954 | 0.027043094 | 0.00761881 | 0.01 to <0.02 |
| Yemen | 0.063773938 | 0.102960854 | 0.036422319 | 0.05 to <0.08 |
| Finland | 0.009262841 | 0.01068195 | 0.008161768 | <0.01 |
| Papua New Guinea | 0.022667279 | 0.043119162 | 0.012959686 | 0.02 to <0.02 |
| Ghana | 0.082874399 | 0.13163788 | 0.051428603 | 0.08 to <0.13 |
| Grenada | 0.01350196 | 0.019194848 | 0.009361939 | 0.01 to <0.02 |
| Costa Rica | 0.041647994 | 0.047732778 | 0.036412909 | 0.04 to <0.05 |
| Saint Kitts and Nevis | 0.017133873 | 0.020571611 | 0.014141565 | 0.02 to <0.02 |
| Vanuatu | 0.008284375 | 0.016822788 | 0.004300938 | <0.01 |
| Lithuania | 0.013710169 | 0.015710938 | 0.011924519 | 0.01 to <0.02 |
| Lesotho | 0.016903961 | 0.03445824 | 0.006880975 | 0.02 to <0.02 |
| Russian Federation | 0.06479676 | 0.068177145 | 0.061879737 | 0.05 to <0.08 |
| Guatemala | 0.149667074 | 0.166552442 | 0.134229687 | 0.13 to <0.88 |
| Czechia | 0.019025269 | 0.02304401 | 0.015891855 | 0.02 to <0.02 |
| Burundi | 0.087550379 | 0.162034766 | 0.046505504 | 0.08 to <0.13 |
| Guinea | 0.474704965 | 0.741465691 | 0.199757863 | 0.13 to <0.88 |

**Supplementary Table S2**: Age-standardized mortality rates of Hepatoblastoma by country and region in 2021

| location | val | upper | lower | val2 |
| --- | --- | --- | --- | --- |
| Democratic People's Republic of Korea | 0.026551564 | 0.059075626 | 0.013071597 | 0.02 to <0.03 |
| Turkmenistan | 0.023024875 | 0.029294266 | 0.017906929 | 0.02 to <0.02 |
| Australia | 0.01676422 | 0.021109984 | 0.013099658 | 0.01 to <0.02 |
| Paraguay | 0.027997137 | 0.04566903 | 0.016798394 | 0.02 to <0.03 |
| Kyrgyzstan | 0.007623186 | 0.010028539 | 0.005698149 | 0.01 to <0.01 |
| Syrian Arab Republic | 0.027767662 | 0.042562216 | 0.013688562 | 0.02 to <0.03 |
| Kiribati | 0.017389503 | 0.039086244 | 0.007839848 | 0.01 to <0.02 |
| Mongolia | 0.181113062 | 0.272693678 | 0.114514682 | 0.06 to <0.25 |
| Central African Republic | 0.060271338 | 0.108273479 | 0.029193691 | 0.06 to <0.25 |
| Papua New Guinea | 0.014712204 | 0.026978828 | 0.007770732 | 0.01 to <0.02 |
| Democratic Republic of the Congo | 0.019646868 | 0.04191739 | 0.009862946 | 0.02 to <0.02 |
| Coted'Ivoire | 0.062345443 | 0.10554405 | 0.037310778 | 0.06 to <0.25 |
| Bahamas | 0.008590663 | 0.011972704 | 0.006249726 | 0.01 to <0.01 |
| Brazil | 0.014500523 | 0.017905187 | 0.011176543 | 0.01 to <0.02 |
| Fiji | 0.013896807 | 0.023612565 | 0.007455915 | 0.01 to <0.02 |
| Cabo Verde | 0.022282646 | 0.035435458 | 0.013830868 | 0.02 to <0.02 |
| Netherlands | 0.005326835 | 0.00629519 | 0.004487691 | <0.01 |
| China | 0.02890754 | 0.040842551 | 0.020726494 | 0.02 to <0.03 |
| Angola | 0.059003734 | 0.121524255 | 0.01926593 | 0.06 to <0.25 |
| Cameroon | 0.062981972 | 0.124413652 | 0.024092605 | 0.06 to <0.25 |
| Slovenia | 0.003318035 | 0.004395323 | 0.002447481 | <0.01 |
| Tajikistan | 0.030484693 | 0.052985596 | 0.016507455 | 0.02 to <0.03 |
| Portugal | 0.009840927 | 0.012556299 | 0.00765822 | 0.01 to <0.01 |
| Marshall Islands | 0.005810335 | 0.013332576 | 0.002373172 | <0.01 |
| Ecuador | 0.013481791 | 0.018242409 | 0.009850865 | 0.01 to <0.02 |
| Monaco | 0.012395293 | 0.018964083 | 0.008107037 | 0.01 to <0.01 |
| Latvia | 0.021230757 | 0.028094025 | 0.016062999 | 0.02 to <0.02 |
| Seychelles | 0.006756077 | 0.00940447 | 0.004686085 | 0.01 to <0.01 |
| Malta | 0.007907389 | 0.010175831 | 0.00581615 | 0.01 to <0.01 |
| New Zealand | 0.016342353 | 0.02208569 | 0.012524952 | 0.01 to <0.02 |
| Niue | 0.021952986 | 0.0467856 | 0.010915852 | 0.02 to <0.02 |
| Belarus | 0.02987902 | 0.044662635 | 0.018940497 | 0.02 to <0.03 |
| Turkey | 0.010566262 | 0.016331956 | 0.007153658 | 0.01 to <0.01 |
| Cyprus | 0.00508401 | 0.007935871 | 0.003150937 | <0.01 |
| Lao People's Democratic Republic | 0.037541438 | 0.057252257 | 0.022855155 | 0.03 to <0.06 |
| Chad | 0.089853302 | 0.140874192 | 0.052362989 | 0.06 to <0.25 |
| Bolivia (Plurinational State of) | 0.029952469 | 0.048687352 | 0.018943398 | 0.02 to <0.03 |
| Austria | 0.011231049 | 0.014667413 | 0.008614865 | 0.01 to <0.01 |
| Algeria | 0.019144541 | 0.030274534 | 0.01175326 | 0.02 to <0.02 |
| United Republic of Tanzania | 0.06760829 | 0.114479109 | 0.038477489 | 0.06 to <0.25 |
| Congo | 0.033247846 | 0.072467087 | 0.01279731 | 0.03 to <0.06 |
| Norway | 0.020193043 | 0.024160944 | 0.016872254 | 0.02 to <0.02 |
| Taiwan (Province of China) | 0.020069306 | 0.024019197 | 0.016724415 | 0.02 to <0.02 |
| Antigua and Barbuda | 0.00751658 | 0.009032438 | 0.006208929 | 0.01 to <0.01 |
| Estonia | 0.026056122 | 0.033798254 | 0.019347127 | 0.02 to <0.03 |
| Cambodia | 0.028723458 | 0.054215856 | 0.014964523 | 0.02 to <0.03 |
| Yemen | 0.025240477 | 0.042532373 | 0.013716846 | 0.02 to <0.03 |
| Somalia | 0.063544489 | 0.128041902 | 0.024891092 | 0.06 to <0.25 |
| Denmark | 0.003194281 | 0.00398216 | 0.002462869 | <0.01 |
| Spain | 0.01490398 | 0.01807093 | 0.012264286 | 0.01 to <0.02 |
| Bulgaria | 0.00850034 | 0.011622701 | 0.006087617 | 0.01 to <0.01 |
| Peru | 0.012347374 | 0.021474208 | 0.006581519 | 0.01 to <0.01 |
| Nauru | 0.018681987 | 0.032921997 | 0.010091089 | 0.02 to <0.02 |
| Tunisia | 0.011893272 | 0.018621697 | 0.00759512 | 0.01 to <0.01 |
| Belize | 0.008628164 | 0.010704421 | 0.006952475 | 0.01 to <0.01 |
| Dominica | 0.017373418 | 0.026993737 | 0.011214079 | 0.01 to <0.02 |
| Micronesia (Federated States of) | 0.006254036 | 0.01376687 | 0.00284391 | 0.01 to <0.01 |
| Andorra | 0.038987417 | 0.061925326 | 0.022619083 | 0.03 to <0.06 |
| Egypt | 0.0567754 | 0.078687698 | 0.042275588 | 0.03 to <0.06 |
| Comoros | 0.050756251 | 0.087321547 | 0.0291803 | 0.03 to <0.06 |
| Gambia | 0.194952496 | 0.290450842 | 0.126643803 | 0.06 to <0.25 |
| Uzbekistan | 0.022821397 | 0.030760035 | 0.016480001 | 0.02 to <0.02 |
| Colombia | 0.017207325 | 0.023644898 | 0.012251602 | 0.01 to <0.02 |
| Ukraine | 0.016824433 | 0.020819261 | 0.012794272 | 0.01 to <0.02 |
| Liberia | 0.07837911 | 0.131518678 | 0.040284982 | 0.06 to <0.25 |
| Ghana | 0.028335435 | 0.050589698 | 0.016186972 | 0.02 to <0.03 |
| Switzerland | 0.007265622 | 0.008933667 | 0.005785201 | 0.01 to <0.01 |
| Uganda | 0.11092685 | 0.202099095 | 0.059602552 | 0.06 to <0.25 |
| Philippines | 0.033254915 | 0.04320133 | 0.02595693 | 0.03 to <0.06 |
| San Marino | 0.007169614 | 0.012742693 | 0.00409471 | 0.01 to <0.01 |
| Albania | 0.055034328 | 0.091399865 | 0.033006868 | 0.03 to <0.06 |
| Greece | 0.022260664 | 0.02643842 | 0.018370009 | 0.02 to <0.02 |
| Iran (Islamic Republic of) | 0.023480657 | 0.038429714 | 0.016162526 | 0.02 to <0.02 |
| Zambia | 0.079268543 | 0.17641458 | 0.035119281 | 0.06 to <0.25 |
| Bahrain | 0.01029472 | 0.014248194 | 0.006776797 | 0.01 to <0.01 |
| United Arab Emirates | 0.011996555 | 0.017798977 | 0.008128159 | 0.01 to <0.01 |
| North Macedonia | 0.011706484 | 0.017778361 | 0.007617269 | 0.01 to <0.01 |
| Guinea | 0.182071155 | 0.279173582 | 0.102543744 | 0.06 to <0.25 |
| Bhutan | 0.057390239 | 0.100011507 | 0.027331809 | 0.03 to <0.06 |
| Sri Lanka | 0.003217075 | 0.005215978 | 0.002052829 | <0.01 |
| Indonesia | 0.021088145 | 0.036787839 | 0.009640606 | 0.02 to <0.02 |
| Jamaica | 0.006241818 | 0.008466035 | 0.00448246 | 0.01 to <0.01 |
| Northern Mariana Islands | 0.003850307 | 0.007136582 | 0.002075805 | <0.01 |
| Afghanistan | 0.066232454 | 0.100225063 | 0.042914665 | 0.06 to <0.25 |
| Samoa | 0.018636757 | 0.046276192 | 0.008133918 | 0.01 to <0.02 |
| Solomon Islands | 0.008981332 | 0.021087288 | 0.00434965 | 0.01 to <0.01 |
| El Salvador | 0.00883881 | 0.013637322 | 0.006050552 | 0.01 to <0.01 |
| Barbados | 0.005851913 | 0.008262267 | 0.004233846 | 0.01 to <0.01 |
| Bosnia and Herzegovina | 0.008070586 | 0.011073239 | 0.005657882 | 0.01 to <0.01 |
| Russian Federation | 0.025110874 | 0.027534621 | 0.022527855 | 0.02 to <0.03 |
| Equatorial Guinea | 0.029106353 | 0.05452614 | 0.014588899 | 0.02 to <0.03 |
| Georgia | 0.005528525 | 0.007208919 | 0.004162511 | <0.01 |
| Republic of Korea | 0.014906594 | 0.023873112 | 0.009316782 | 0.01 to <0.02 |
| Botswana | 0.025248033 | 0.047772057 | 0.013716387 | 0.02 to <0.03 |
| Madagascar | 0.027250497 | 0.053316698 | 0.013628959 | 0.02 to <0.03 |
| Guinea-Bissau | 0.058040124 | 0.087673098 | 0.032695514 | 0.06 to <0.25 |
| Puerto Rico | 0.004763068 | 0.006115212 | 0.003738229 | <0.01 |
| Cuba | 0.004575353 | 0.005912951 | 0.00341466 | <0.01 |
| Lithuania | 0.020317477 | 0.026218983 | 0.015719748 | 0.02 to <0.02 |
| Nigeria | 0.040469774 | 0.055130496 | 0.026131807 | 0.03 to <0.06 |
| France | 0.018440111 | 0.022506518 | 0.014873581 | 0.01 to <0.02 |
| Palau | 0.014393171 | 0.029940126 | 0.006755019 | 0.01 to <0.02 |
| Sudan | 0.055185778 | 0.087927417 | 0.03449248 | 0.03 to <0.06 |
| Georgia | 0.021192726 | 0.024091553 | 0.018797066 | 0.02 to <0.02 |
| Kuwait | 0.003671956 | 0.004733957 | 0.002844553 | <0.01 |
| Burundi | 0.026835212 | 0.060426996 | 0.010826909 | 0.02 to <0.03 |
| Lesotho | 0.029998606 | 0.085311257 | 0.011528906 | 0.02 to <0.03 |
| Sao Tome and Principe | 0.009326974 | 0.02254459 | 0.003884872 | 0.01 to <0.01 |
| Guyana | 0.008774933 | 0.011367053 | 0.006440469 | 0.01 to <0.01 |
| Sweden | 0.008709821 | 0.011078036 | 0.006834502 | 0.01 to <0.01 |
| Slovakia | 0.008111919 | 0.014602974 | 0.004605526 | 0.01 to <0.01 |
| Belgium | 0.008772581 | 0.011516709 | 0.006629783 | 0.01 to <0.01 |
| Luxembourg | 0.008500785 | 0.010414224 | 0.006899244 | 0.01 to <0.01 |
| Lebanon | 0.009106362 | 0.018084717 | 0.004743551 | 0.01 to <0.01 |
| Saint Kitts and Nevis | 0.009255736 | 0.012386415 | 0.006907022 | 0.01 to <0.01 |
| Mauritania | 0.075785411 | 0.123059965 | 0.037249662 | 0.06 to <0.25 |
| Republic of Moldova | 0.069698532 | 0.092691333 | 0.053553316 | 0.06 to <0.25 |
| Rwanda | 0.04046711 | 0.076457569 | 0.021970929 | 0.03 to <0.06 |
| United Kingdom | 0.016625867 | 0.018058502 | 0.015037952 | 0.01 to <0.02 |
| Nicaragua | 0.020819993 | 0.031231834 | 0.013669289 | 0.02 to <0.02 |
| Uruguay | 0.003075862 | 0.004102472 | 0.002200273 | <0.01 |
| Costa Rica | 0.022107516 | 0.027955877 | 0.017275738 | 0.02 to <0.02 |
| Nepal | 0.0261976 | 0.038800241 | 0.015902906 | 0.02 to <0.03 |
| Tonga | 0.040912636 | 0.089578452 | 0.019167354 | 0.03 to <0.06 |
| Armenia | 0.021798257 | 0.029359518 | 0.016519982 | 0.02 to <0.02 |
| Panama | 0.026634953 | 0.033487699 | 0.020685916 | 0.02 to <0.03 |
| Gabon | 0.037812441 | 0.067191621 | 0.0174183 | 0.03 to <0.06 |
| Ireland | 0.005393363 | 0.006561679 | 0.00425802 | <0.01 |
| Malaysia | 0.013754114 | 0.019897293 | 0.008888252 | 0.01 to <0.02 |
| Guatemala | 0.038935056 | 0.049926116 | 0.030113867 | 0.03 to <0.06 |
| Zimbabwe | 0.066009948 | 0.104649685 | 0.036376549 | 0.06 to <0.25 |
| Israel | 0.006029271 | 0.007478366 | 0.004729107 | 0.01 to <0.01 |
| Oman | 0.013826984 | 0.021613195 | 0.008724758 | 0.01 to <0.02 |
| Saint Vincent and the Grenadines | 0.008723897 | 0.011152876 | 0.006720577 | 0.01 to <0.01 |
| Vanuatu | 0.005889025 | 0.011406861 | 0.002624048 | 0.01 to <0.01 |
| Jordan | 0.009292473 | 0.014598585 | 0.006185299 | 0.01 to <0.01 |
| Hungary | 0.003425914 | 0.004468072 | 0.002530498 | <0.01 |
| Maldives | 0.012394085 | 0.019531348 | 0.007540497 | 0.01 to <0.01 |
| Iraq | 0.022858729 | 0.03678052 | 0.014231138 | 0.02 to <0.02 |
| Croatia | 0.003008146 | 0.004478449 | 0.002072113 | <0.01 |
| Finland | 0.005242933 | 0.006652866 | 0.004080678 | <0.01 |
| Mali | 0.250169111 | 0.375655562 | 0.146218495 | 0.06 to <0.25 |
| Namibia | 0.033277317 | 0.051672778 | 0.020825622 | 0.03 to <0.06 |
| Sierra Leone | 0.079637773 | 0.121249598 | 0.044379699 | 0.06 to <0.25 |
| Myanmar | 0.024756344 | 0.048616302 | 0.009521195 | 0.02 to <0.03 |
| Argentina | 0.005264423 | 0.006469179 | 0.004214787 | <0.01 |
| Libya | 0.086481166 | 0.129081363 | 0.048383928 | 0.06 to <0.25 |
| Niger | 0.056878687 | 0.093205119 | 0.033976402 | 0.03 to <0.06 |
| United States Virgin Islands | 0.00330936 | 0.006574604 | 0.001596885 | <0.01 |
| Ethiopia | 0.032256598 | 0.066281179 | 0.016572904 | 0.02 to <0.03 |
| Honduras | 0.01390618 | 0.023364881 | 0.007977546 | 0.01 to <0.02 |
| Dominican Republic | 0.012306791 | 0.017695334 | 0.007910596 | 0.01 to <0.01 |
| Haiti | 0.017072123 | 0.032124019 | 0.008514016 | 0.01 to <0.02 |
| Eritrea | 0.042161052 | 0.09584375 | 0.018016703 | 0.03 to <0.06 |
| Tokelau | 0.008571518 | 0.025568786 | 0.003301718 | 0.01 to <0.01 |
| Mexico | 0.029517507 | 0.039364712 | 0.022194538 | 0.02 to <0.03 |
| Bangladesh | 0.025826404 | 0.036531504 | 0.017019374 | 0.02 to <0.03 |
| Kenya | 0.034660764 | 0.049482527 | 0.024691006 | 0.03 to <0.06 |
| Chile | 0.003729854 | 0.004789733 | 0.002857666 | <0.01 |
| Japan | 0.01262136 | 0.013920299 | 0.011239769 | 0.01 to <0.02 |
| Eswatini | 0.045740463 | 0.111804457 | 0.017738047 | 0.03 to <0.06 |
| India | 0.024732885 | 0.034156864 | 0.017854413 | 0.02 to <0.02 |
| South Africa | 0.020777396 | 0.027219062 | 0.015087848 | 0.02 to <0.02 |
| Djibouti | 0.041972752 | 0.085232312 | 0.019447409 | 0.03 to <0.06 |
| Azerbaijan | 0.041966716 | 0.074352507 | 0.022932293 | 0.03 to <0.06 |
| Thailand | 0.03254846 | 0.043754252 | 0.023610491 | 0.02 to <0.03 |
| Tuvalu | 0.007998547 | 0.015421108 | 0.004364905 | 0.01 to <0.01 |
| Iceland | 0.00749255 | 0.009795175 | 0.005886569 | 0.01 to <0.01 |
| Venezuela (Bolivarian Republic of) | 0.036634806 | 0.049720101 | 0.026535445 | 0.03 to <0.06 |
| Serbia | 0.00622886 | 0.011227069 | 0.003158637 | 0.01 to <0.01 |
| Brunei Darussalam | 0.017703454 | 0.02730156 | 0.011493499 | 0.01 to <0.02 |
| Viet Nam | 0.054984329 | 0.085696032 | 0.034020208 | 0.03 to <0.06 |
| Grenada | 0.009866602 | 0.012529212 | 0.00747658 | 0.01 to <0.01 |
| Qatar | 0.015628315 | 0.024335325 | 0.009818648 | 0.01 to <0.02 |
| Togo | 0.028225143 | 0.047255269 | 0.016245077 | 0.02 to <0.03 |
| Suriname | 0.013056695 | 0.020006075 | 0.008241968 | 0.01 to <0.02 |
| Palestine | 0.021180043 | 0.032492523 | 0.013887849 | 0.02 to <0.02 |
| Montenegro | 0.010600896 | 0.017106292 | 0.005768896 | 0.01 to <0.01 |
| Germany | 0.010428771 | 0.013058262 | 0.008315701 | 0.01 to <0.01 |
| Morocco | 0.004348087 | 0.00776119 | 0.002334202 | <0.01 |
| Mozambique | 0.036993689 | 0.097032092 | 0.01500432 | 0.03 to <0.06 |
| Senegal | 0.035963171 | 0.052451822 | 0.023937413 | 0.03 to <0.06 |
| Trinidad and Tobago | 0.007093096 | 0.009534085 | 0.005173331 | 0.01 to <0.01 |
| Kazakhstan | 0.014815234 | 0.018744816 | 0.011460155 | 0.01 to <0.02 |
| American Samoa | 0.00886171 | 0.017828422 | 0.004366429 | 0.01 to <0.01 |
| Canada | 0.018653693 | 0.02270402 | 0.015187635 | 0.02 to <0.02 |
| Singapore | 0.005838927 | 0.007264924 | 0.004682019 | <0.01 |
| Bermuda | 0.003313285 | 0.004422279 | 0.002364679 | <0.01 |
| Poland | 0.00351518 | 0.004119839 | 0.002922529 | <0.01 |
| Czechia | 0.002690815 | 0.003658303 | 0.001921648 | <0.01 |
| Timor-Leste | 0.020389747 | 0.034743547 | 0.009692456 | 0.02 to <0.02 |
| South Sudan | 0.12803886 | 0.213767382 | 0.052656711 | 0.06 to <0.25 |
| United States of America | 0.021903823 | 0.024325967 | 0.019514785 | 0.02 to <0.02 |
| Benin | 0.098255343 | 0.159801946 | 0.055050051 | 0.06 to <0.25 |
| Pakistan | 0.080798716 | 0.113240415 | 0.055367034 | 0.06 to <0.25 |
| Saint Lucia | 0.006576626 | 0.008900421 | 0.004899147 | 0.01 to <0.01 |
| Cook Islands | 0.0045598 | 0.01011265 | 0.002239153 | <0.01 |
| Mauritius | 0.001348292 | 0.001551702 | 0.001120817 | <0.01 |
| Malawi | 0.099722163 | 0.229670968 | 0.043300848 | 0.06 to <0.25 |
| Saudi Arabia | 0.010339167 | 0.017667919 | 0.006289602 | 0.01 to <0.01 |
| Burkina Faso | 0.139650019 | 0.238866144 | 0.068496958 | 0.06 to <0.25 |
| Greenland | 0.029290742 | 0.046566671 | 0.017597524 | 0.02 to <0.03 |
| Guam | 0.016297291 | 0.023529567 | 0.011143374 | 0.01 to <0.02 |
| Italy | 0.008660479 | 0.010310036 | 0.007040377 | 0.01 to <0.01 |

**Supplementary Table S3**: Age-standardized disability rates of Hepatoblastoma by country and region in 1990

| location | val | upper | lower | val2 |
| --- | --- | --- | --- | --- |
| Papua New Guinea | 2.00947739 | 3.828703195 | 1.154789262 | 1.43 to <2.02 |
| Israel | 1.157536188 | 1.336949559 | 0.988392025 | 0.9 to <1.43 |
| Argentina | 0.412616557 | 0.48198881 | 0.349287006 | <0.9 |
| Maldives | 3.247765962 | 5.609566907 | 1.128832258 | 3.21 to <4.6 |
| Montenegro | 3.168730756 | 4.431251618 | 2.118132215 | 2.02 to <3.21 |
| Mongolia | 77.63883773 | 120.7829144 | 47.05047151 | 11.02 to <77.64 |
| Cuba | 0.824729808 | 0.999441936 | 0.700647072 | <0.9 |
| Angola | 21.30910011 | 41.05102271 | 7.542828037 | 11.02 to <77.64 |
| Kenya | 5.015803162 | 6.940893898 | 3.009232354 | 4.6 to <6.63 |
| Botswana | 1.293328844 | 2.344132682 | 0.667674931 | 0.9 to <1.43 |
| Bolivia (Plurinational State of) | 7.157085861 | 11.40511416 | 3.556958043 | 6.63 to <11.02 |
| Coted'Ivoire | 13.46935769 | 22.31450836 | 7.545899986 | 11.02 to <77.64 |
| Venezuela (Bolivarian Republic of) | 3.21592923 | 3.443363677 | 2.985384551 | 3.21 to <4.6 |
| Tajikistan | 5.44731302 | 8.949989541 | 2.296763999 | 4.6 to <6.63 |
| Andorra | 8.5145297 | 14.87598168 | 3.59673917 | 6.63 to <11.02 |
| Kuwait | 3.390829527 | 4.169015817 | 2.776635179 | 3.21 to <4.6 |
| American Samoa | 0.512540375 | 0.928163887 | 0.288673191 | <0.9 |
| Italy | 2.350290917 | 2.487083694 | 2.228908258 | 2.02 to <3.21 |
| Russian Federation | 5.688140553 | 5.97485597 | 5.427874651 | 4.6 to <6.63 |
| Central African Republic | 12.16421777 | 19.84044651 | 6.448215921 | 11.02 to <77.64 |
| Myanmar | 4.727129713 | 9.9887071 | 0.839289606 | 4.6 to <6.63 |
| Sierra Leone | 22.12031138 | 33.467761 | 11.57328998 | 11.02 to <77.64 |
| Samoa | 3.122953757 | 5.360820021 | 1.813673811 | 2.02 to <3.21 |
| Puerto Rico | 0.804967793 | 0.981962108 | 0.658103375 | <0.9 |
| Chile | 0.397431148 | 0.518501885 | 0.312043346 | <0.9 |
| Madagascar | 6.233199904 | 10.03133682 | 3.780623054 | 4.6 to <6.63 |
| Poland | 0.38043628 | 0.426147209 | 0.343860258 | <0.9 |
| Ukraine | 3.957959626 | 5.0583505 | 3.129420878 | 3.21 to <4.6 |
| Dominica | 0.747380322 | 1.149640995 | 0.476311518 | <0.9 |
| Austria | 1.481556032 | 1.719291127 | 1.275283942 | 1.43 to <2.02 |
| Ecuador | 4.484692953 | 5.44023648 | 3.703816767 | 3.21 to <4.6 |
| Yemen | 5.63385517 | 9.097601864 | 3.228743416 | 4.6 to <6.63 |
| Solomon Islands | 1.344876801 | 3.160025619 | 0.501292997 | 0.9 to <1.43 |
| Romania | 3.313544553 | 4.206613305 | 2.627741404 | 3.21 to <4.6 |
| Saint Kitts and Nevis | 1.508143827 | 1.809789025 | 1.245150391 | 1.43 to <2.02 |
| Gambia | 31.95445761 | 47.19801894 | 21.2954856 | 11.02 to <77.64 |
| Uruguay | 0.307118543 | 0.38151809 | 0.248398092 | <0.9 |
| Dominican Republic | 2.24820072 | 3.354948996 | 1.228985475 | 2.02 to <3.21 |
| Luxembourg | 1.414222818 | 1.587520352 | 1.252776166 | 0.9 to <1.43 |
| Congo | 10.58557756 | 18.423814 | 5.399992458 | 6.63 to <11.02 |
| Togo | 5.475952574 | 8.236965445 | 3.383976616 | 4.6 to <6.63 |
| Philippines | 8.24526035 | 10.77236773 | 5.55064396 | 6.63 to <11.02 |
| Brazil | 2.788699012 | 3.297427945 | 2.406577161 | 2.02 to <3.21 |
| Lebanon | 2.424022023 | 3.616899128 | 1.560220084 | 2.02 to <3.21 |
| Malawi | 22.23471279 | 33.52052213 | 13.87491777 | 11.02 to <77.64 |
| Lesotho | 1.482124292 | 3.035498739 | 0.60370394 | 1.43 to <2.02 |
| Namibia | 2.314723604 | 3.620710588 | 1.291712496 | 2.02 to <3.21 |
| Bermuda | 0.790593421 | 1.012798329 | 0.594119788 | <0.9 |
| Turkmenistan | 7.923238375 | 9.095550958 | 6.676316584 | 6.63 to <11.02 |
| Ghana | 7.360914444 | 11.60135689 | 4.57059714 | 6.63 to <11.02 |
| Peru | 3.822276201 | 5.587620829 | 2.326648755 | 3.21 to <4.6 |
| Sri Lanka | 0.66274634 | 0.880794194 | 0.485009053 | <0.9 |
| Libya | 5.799396431 | 8.256850407 | 3.887781348 | 4.6 to <6.63 |
| Mauritius | 1.326586681 | 1.473889236 | 1.204343918 | 0.9 to <1.43 |
| Democratic Republic of the Congo | 5.841362778 | 11.57573985 | 2.429157888 | 4.6 to <6.63 |
| Cook Islands | 1.450833384 | 2.476632996 | 0.833713082 | 1.43 to <2.02 |
| Uzbekistan | 3.59502098 | 4.922402805 | 2.539268451 | 3.21 to <4.6 |
| Sudan | 10.00557233 | 16.58400381 | 5.11679923 | 6.63 to <11.02 |
| Belgium | 1.182841826 | 1.381528609 | 1.004257707 | 0.9 to <1.43 |
| Malta | 0.706682623 | 0.845832844 | 0.55756983 | <0.9 |
| Tonga | 4.497255631 | 8.883647457 | 2.342088233 | 3.21 to <4.6 |
| South Africa | 2.077704886 | 2.797143154 | 1.363628329 | 2.02 to <3.21 |
| Cyprus | 1.218657096 | 1.91715644 | 0.760921946 | 0.9 to <1.43 |
| Grenada | 1.189079906 | 1.691854783 | 0.821347175 | 0.9 to <1.43 |
| Paraguay | 3.241607452 | 4.759588872 | 2.191858798 | 3.21 to <4.6 |
| San Marino | 1.549328011 | 2.372612556 | 0.985828993 | 1.43 to <2.02 |
| Serbia | 3.324716619 | 5.652349253 | 1.691757372 | 3.21 to <4.6 |
| Georgia | 1.431539537 | 1.52217492 | 1.341796732 | 0.9 to <1.43 |
| Canada | 1.090210909 | 1.220925297 | 0.972606405 | 0.9 to <1.43 |
| Morocco | 0.847850379 | 1.309151567 | 0.533074567 | <0.9 |
| Guinea | 42.22586708 | 66.09514827 | 17.80038228 | 11.02 to <77.64 |
| Thailand | 11.02552252 | 14.74256654 | 8.057146241 | 11.02 to <77.64 |
| Tokelau | 0.82437563 | 1.868096756 | 0.368437133 | <0.9 |
| Netherlands | 0.688550826 | 0.773150827 | 0.611921286 | <0.9 |
| Colombia | 3.208710897 | 3.819055204 | 2.70726101 | 2.02 to <3.21 |
| Guyana | 1.424873229 | 1.771965138 | 1.123911353 | 0.9 to <1.43 |
| Vanuatu | 0.732562312 | 1.493812181 | 0.380348275 | <0.9 |
| Equatorial Guinea | 3.755680857 | 7.965447118 | 1.418437009 | 3.21 to <4.6 |
| Palestine | 4.334731463 | 6.397257882 | 2.732088873 | 3.21 to <4.6 |
| Greenland | 4.642447658 | 6.520067712 | 3.325193548 | 4.6 to <6.63 |
| China | 12.59737153 | 15.41788616 | 10.48147433 | 11.02 to <77.64 |
| Denmark | 0.784067562 | 0.894309627 | 0.691240243 | <0.9 |
| Brunei Darussalam | 2.241798773 | 3.296753577 | 1.504938425 | 2.02 to <3.21 |
| Afghanistan | 12.46267544 | 22.31333178 | 4.138748429 | 11.02 to <77.64 |
| Slovakia | 1.941268281 | 2.93528606 | 1.258581528 | 1.43 to <2.02 |
| Timor-Leste | 4.9790743 | 8.967164435 | 1.089877463 | 4.6 to <6.63 |
| Norway | 1.188589848 | 1.282531284 | 1.092770589 | 0.9 to <1.43 |
| Democratic People's Republic of Korea | 9.072518259 | 18.15457304 | 4.311876494 | 6.63 to <11.02 |
| Mozambique | 8.866869689 | 13.57720263 | 4.878129761 | 6.63 to <11.02 |
| Guinea-Bissau | 16.78116461 | 28.20595268 | 8.390345838 | 11.02 to <77.64 |
| Eswatini | 2.591542237 | 4.187466402 | 1.359196709 | 2.02 to <3.21 |
| Guam | 0.481791422 | 0.671820594 | 0.357282322 | <0.9 |
| Costa Rica | 3.680428842 | 4.229874603 | 3.218720912 | 3.21 to <4.6 |
| United States of America | 1.584773341 | 1.623749061 | 1.545598551 | 1.43 to <2.02 |
| Oman | 1.940628531 | 3.071448108 | 1.141788876 | 1.43 to <2.02 |
| Gabon | 6.44717432 | 11.43719338 | 3.382164202 | 4.6 to <6.63 |
| Tuvalu | 1.784521202 | 3.165948918 | 0.906911862 | 1.43 to <2.02 |
| Haiti | 2.618339852 | 5.225569723 | 0.962485022 | 2.02 to <3.21 |
| Albania | 18.39248782 | 26.39706861 | 12.24402216 | 11.02 to <77.64 |
| Bhutan | 6.729049703 | 12.48701867 | 2.795412801 | 6.63 to <11.02 |
| Rwanda | 9.148069958 | 14.7386376 | 4.688077421 | 6.63 to <11.02 |
| Armenia | 4.180575139 | 5.297089789 | 3.362263514 | 3.21 to <4.6 |
| Finland | 0.817639384 | 0.942489848 | 0.720399587 | <0.9 |
| Liberia | 23.326356 | 39.11860864 | 8.820787777 | 11.02 to <77.64 |
| Slovenia | 1.699280594 | 1.995131886 | 1.447393919 | 1.43 to <2.02 |
| Seychelles | 1.357698143 | 2.095059435 | 0.926742103 | 0.9 to <1.43 |
| United States Virgin Islands | 0.528482761 | 0.776280204 | 0.339234485 | <0.9 |
| Zimbabwe | 4.934966903 | 8.177100633 | 2.638365785 | 4.6 to <6.63 |
| Japan | 2.57683743 | 2.699173843 | 2.47699694 | 2.02 to <3.21 |
| Bangladesh | 5.939243252 | 9.945152719 | 2.237218093 | 4.6 to <6.63 |
| Portugal | 2.039719802 | 2.41349197 | 1.664828724 | 2.02 to <3.21 |
| Viet Nam | 14.65360804 | 20.81629619 | 9.719734068 | 11.02 to <77.64 |
| Mali | 35.14913448 | 53.65175117 | 15.50785415 | 11.02 to <77.64 |
| Taiwan (Province of China) | 3.411678044 | 3.860468818 | 3.016203314 | 3.21 to <4.6 |
| Jamaica | 0.993970175 | 1.28760167 | 0.723768418 | 0.9 to <1.43 |
| Bosnia and Herzegovina | 2.3897222 | 3.244540355 | 1.704306585 | 2.02 to <3.21 |
| Algeria | 3.333300875 | 4.810412268 | 2.390872549 | 3.21 to <4.6 |
| Republic of Korea | 6.63291636 | 9.620077195 | 4.008989632 | 4.6 to <6.63 |
| Monaco | 2.240922246 | 3.764750302 | 1.294794988 | 2.02 to <3.21 |
| El Salvador | 2.903028955 | 3.626915714 | 2.232583961 | 2.02 to <3.21 |
| France | 1.90910209 | 2.157387728 | 1.678443107 | 1.43 to <2.02 |
| Qatar | 2.609925471 | 3.738803157 | 1.757474388 | 2.02 to <3.21 |
| Belarus | 1.196557893 | 1.55775083 | 0.930561655 | 0.9 to <1.43 |
| Bulgaria | 3.924130365 | 5.053197646 | 2.969151804 | 3.21 to <4.6 |
| Singapore | 2.734957839 | 3.295032733 | 2.266069241 | 2.02 to <3.21 |
| Spain | 2.151134128 | 2.464065397 | 1.886349911 | 2.02 to <3.21 |
| Burundi | 7.706033935 | 14.22103713 | 4.118238595 | 6.63 to <11.02 |
| Germany | 1.347674382 | 1.620997274 | 1.137993691 | 0.9 to <1.43 |
| Somalia | 10.58379939 | 18.19550926 | 5.43948474 | 6.63 to <11.02 |
| Sweden | 1.469285122 | 1.690739934 | 1.270084074 | 1.43 to <2.02 |
| Saint Lucia | 0.949611148 | 1.196682879 | 0.728338128 | 0.9 to <1.43 |
| Fiji | 0.733275025 | 1.462613158 | 0.394056027 | <0.9 |
| Croatia | 1.263255788 | 1.66710689 | 0.924655822 | 0.9 to <1.43 |
| Benin | 17.93767663 | 28.04063531 | 11.26368121 | 11.02 to <77.64 |
| Nauru | 2.033602645 | 3.41863556 | 1.221128885 | 2.02 to <3.21 |
| Georgia | 2.140656941 | 2.533707445 | 1.761947918 | 2.02 to <3.21 |
| Antigua and Barbuda | 0.780212808 | 0.97980705 | 0.612277479 | <0.9 |
| South Sudan | 10.83401007 | 18.3890979 | 4.370454886 | 6.63 to <11.02 |
| Mauritania | 15.60322324 | 35.59924737 | 4.172399397 | 11.02 to <77.64 |
| Guatemala | 13.11975773 | 14.60525674 | 11.75877489 | 11.02 to <77.64 |
| India | 4.036018805 | 5.452480998 | 2.536421348 | 3.21 to <4.6 |
| Djibouti | 6.220461051 | 11.576009 | 3.574074803 | 4.6 to <6.63 |
| Greece | 1.721701324 | 1.879796816 | 1.578589172 | 1.43 to <2.02 |
| Bahrain | 2.092746617 | 2.854622941 | 1.530181092 | 2.02 to <3.21 |
| United Republic of Tanzania | 11.02157977 | 16.16189225 | 6.901020358 | 6.63 to <11.02 |
| Honduras | 3.503614675 | 5.058821102 | 2.392698202 | 3.21 to <4.6 |
| Niger | 19.26083123 | 30.37882023 | 11.44506259 | 11.02 to <77.64 |
| Saudi Arabia | 4.651736853 | 8.11381791 | 2.865266975 | 4.6 to <6.63 |
| Saint Vincent and the Grenadines | 1.757696351 | 2.215265201 | 1.380285388 | 1.43 to <2.02 |
| Egypt | 11.59470007 | 16.69240546 | 7.097706741 | 11.02 to <77.64 |
| Australia | 1.102442308 | 1.244431901 | 0.972481139 | 0.9 to <1.43 |
| Latvia | 1.531126217 | 1.792026655 | 1.294757017 | 1.43 to <2.02 |
| Comoros | 7.358829221 | 11.73653149 | 3.901175376 | 6.63 to <11.02 |
| Czechia | 1.674170077 | 2.030744595 | 1.395877465 | 1.43 to <2.02 |
| Cambodia | 8.881779536 | 15.50803988 | 4.701339851 | 6.63 to <11.02 |
| Suriname | 1.551966539 | 2.20546454 | 1.033932487 | 1.43 to <2.02 |
| Bahamas | 1.298927638 | 1.588026064 | 1.048840582 | 0.9 to <1.43 |
| Switzerland | 1.970059414 | 2.288837585 | 1.684318615 | 1.43 to <2.02 |
| Azerbaijan | 5.666130968 | 9.931061445 | 2.890022407 | 4.6 to <6.63 |
| Kiribati | 3.120984566 | 5.401068139 | 1.737271876 | 2.02 to <3.21 |
| Estonia | 1.87035971 | 2.271084094 | 1.506700853 | 1.43 to <2.02 |
| Iraq | 4.886080884 | 6.791577653 | 3.226998364 | 4.6 to <6.63 |
| Syrian Arab Republic | 5.356207486 | 7.219828324 | 3.450235213 | 4.6 to <6.63 |
| Burkina Faso | 19.55669976 | 32.57568651 | 11.6506659 | 11.02 to <77.64 |
| Iceland | 0.816574274 | 0.97056186 | 0.68440506 | <0.9 |
| Pakistan | 7.543673804 | 11.11476691 | 4.35363546 | 6.63 to <11.02 |
| Nepal | 4.357902599 | 7.196394536 | 1.523449985 | 3.21 to <4.6 |
| Nicaragua | 7.320628224 | 9.544700611 | 5.603304894 | 6.63 to <11.02 |
| Lao People's Democratic Republic | 10.17491511 | 17.59139498 | 3.069716069 | 6.63 to <11.02 |
| Cameroon | 10.65101468 | 17.05242332 | 5.414302374 | 6.63 to <11.02 |
| Marshall Islands | 0.456950565 | 1.035523304 | 0.195605448 | <0.9 |
| Niue | 1.358741985 | 2.668014298 | 0.642631541 | 0.9 to <1.43 |
| Barbados | 0.795592856 | 0.964539241 | 0.635938135 | <0.9 |
| Sao Tome and Principe | 4.983598158 | 7.460448621 | 3.151083313 | 4.6 to <6.63 |
| Trinidad and Tobago | 1.173253688 | 1.382853281 | 0.988587504 | 0.9 to <1.43 |
| Indonesia | 3.251083865 | 5.510137168 | 1.065676856 | 3.21 to <4.6 |
| Mexico | 4.549909441 | 5.135033871 | 4.09597155 | 3.21 to <4.6 |
| Nigeria | 6.167713617 | 8.395421811 | 3.849779231 | 4.6 to <6.63 |
| Zambia | 17.85740421 | 25.61560484 | 11.86291266 | 11.02 to <77.64 |
| Uganda | 16.21484054 | 23.48943218 | 10.41935672 | 11.02 to <77.64 |
| Kazakhstan | 5.235336119 | 5.985235888 | 4.585463933 | 4.6 to <6.63 |
| Tunisia | 3.372778382 | 4.799670048 | 2.129239035 | 3.21 to <4.6 |
| Northern Mariana Islands | 0.308918714 | 0.541637078 | 0.170370027 | <0.9 |
| Turkey | 3.004061389 | 4.459164107 | 2.08380936 | 2.02 to <3.21 |
| Eritrea | 5.360606006 | 9.501853557 | 2.776335154 | 4.6 to <6.63 |
| Hungary | 1.235256581 | 1.513519863 | 1.008540437 | 0.9 to <1.43 |
| Belize | 2.269854168 | 2.808820061 | 1.885152011 | 2.02 to <3.21 |
| Iran (Islamic Republic of) | 5.606148289 | 7.179429067 | 4.169467031 | 4.6 to <6.63 |
| United Kingdom | 1.062891732 | 1.107344673 | 1.020179511 | 0.9 to <1.43 |
| Ethiopia | 7.098829747 | 11.76016445 | 3.544523979 | 6.63 to <11.02 |
| Micronesia (Federated States of) | 1.117278635 | 1.983449881 | 0.592387935 | 0.9 to <1.43 |
| Cabo Verde | 5.744850432 | 8.898586995 | 2.661751526 | 4.6 to <6.63 |
| Lithuania | 1.210864921 | 1.386696723 | 1.05250149 | 0.9 to <1.43 |
| New Zealand | 1.560184961 | 1.885489056 | 1.28487955 | 1.43 to <2.02 |
| Kyrgyzstan | 8.287632622 | 11.27854136 | 6.036478486 | 6.63 to <11.02 |
| Malaysia | 1.91365362 | 3.060327671 | 1.122312886 | 1.43 to <2.02 |
| Ireland | 0.768890072 | 0.89384726 | 0.660103752 | <0.9 |
| Chad | 11.29855468 | 18.1904106 | 6.768142129 | 11.02 to <77.64 |
| Palau | 1.55544649 | 3.146336567 | 0.696151162 | 1.43 to <2.02 |
| Panama | 3.78181661 | 4.427205564 | 3.12500655 | 3.21 to <4.6 |
| North Macedonia | 8.649688108 | 11.09943076 | 5.964741162 | 6.63 to <11.02 |
| Senegal | 9.523841577 | 14.28547321 | 5.632983635 | 6.63 to <11.02 |
| Republic of Moldova | 12.98581786 | 15.10433443 | 11.20443245 | 11.02 to <77.64 |
| United Arab Emirates | 1.948203916 | 3.211932996 | 1.095299646 | 1.43 to <2.02 |

**Supplementary Table S4**: Age-standardized disability rates of Hepatoblastoma by country and region in 2021

| location | val | upper | lower | val2 |
| --- | --- | --- | --- | --- |
| Bulgaria | 0.743748794 | 1.018235035 | 0.533885902 | 0.52 to <0.77 |
| Haiti | 1.501760209 | 2.838013265 | 0.747003714 | 1.12 to <1.66 |
| Germany | 0.936560455 | 1.166134329 | 0.745097831 | 0.77 to <1.12 |
| Singapore | 0.520087036 | 0.645030649 | 0.41687205 | 0.52 to <0.77 |
| Belarus | 2.644360739 | 3.967359003 | 1.670834243 | 2.18 to <2.86 |
| Costa Rica | 1.942523008 | 2.461018901 | 1.515034552 | 1.66 to <2.18 |
| Myanmar | 2.178971128 | 4.286293104 | 0.838171765 | 1.66 to <2.18 |
| Seychelles | 0.597241878 | 0.82824313 | 0.41552821 | 0.52 to <0.77 |
| Mali | 22.26787654 | 33.42254891 | 13.036611 | 5.12 to <22.27 |
| Ukraine | 1.49380389 | 1.8483429 | 1.139424793 | 1.12 to <1.66 |
| United States of America | 1.958191911 | 2.179519558 | 1.747700289 | 1.66 to <2.18 |
| Jordan | 0.817829343 | 1.290002162 | 0.544865421 | 0.77 to <1.12 |
| United Arab Emirates | 1.043917656 | 1.54011504 | 0.70569092 | 0.77 to <1.12 |
| Czechia | 0.236445004 | 0.322866062 | 0.16811335 | <0.52 |
| Armenia | 1.906844428 | 2.576933508 | 1.441544132 | 1.66 to <2.18 |
| Oman | 1.225157779 | 1.90890702 | 0.772475788 | 1.12 to <1.66 |
| Nauru | 1.650915552 | 2.920657415 | 0.889395908 | 1.12 to <1.66 |
| Austria | 1.007360211 | 1.307503288 | 0.772777928 | 0.77 to <1.12 |
| Luxembourg | 0.765740744 | 0.937540627 | 0.622367886 | 0.52 to <0.77 |
| Panama | 2.346005344 | 2.960589162 | 1.822358499 | 2.18 to <2.86 |
| Coted'Ivoire | 5.528548696 | 9.314416238 | 3.308006764 | 5.12 to <22.27 |
| Trinidad and Tobago | 0.623780503 | 0.839195037 | 0.454714382 | 0.52 to <0.77 |
| Tajikistan | 2.681740912 | 4.64984489 | 1.454673723 | 2.18 to <2.86 |
| Samoa | 1.655740569 | 4.106772118 | 0.723278481 | 1.12 to <1.66 |
| Sierra Leone | 7.118914146 | 10.84454166 | 3.969247674 | 5.12 to <22.27 |
| Democratic People's Republic of Korea | 2.339484562 | 5.215832216 | 1.150144814 | 2.18 to <2.86 |
| Iraq | 2.007641881 | 3.243819568 | 1.254350335 | 1.66 to <2.18 |
| Belize | 0.76083438 | 0.945225617 | 0.612329062 | 0.52 to <0.77 |
| Poland | 0.309381171 | 0.363634307 | 0.257074694 | <0.52 |
| Timor-Leste | 1.792723145 | 3.067216656 | 0.845794429 | 1.66 to <2.18 |
| Angola | 5.185771212 | 10.67308834 | 1.686773551 | 5.12 to <22.27 |
| United Kingdom | 1.487553822 | 1.620926255 | 1.34254671 | 1.12 to <1.66 |
| Chad | 7.983906747 | 12.52601873 | 4.670490995 | 5.12 to <22.27 |
| Botswana | 2.206844401 | 4.171492454 | 1.201836585 | 2.18 to <2.86 |
| Kenya | 3.058255743 | 4.364737176 | 2.178472068 | 2.86 to <5.12 |
| Georgia | 0.484986528 | 0.633203056 | 0.364950902 | <0.52 |
| Australia | 1.502100898 | 1.887085351 | 1.176363868 | 1.12 to <1.66 |
| Iceland | 0.669999112 | 0.87833824 | 0.527638179 | 0.52 to <0.77 |
| Colombia | 1.502954177 | 2.06414332 | 1.068144582 | 1.12 to <1.66 |
| Bhutan | 5.065693805 | 8.827816436 | 2.411832769 | 2.86 to <5.12 |
| Latvia | 1.872135667 | 2.474445124 | 1.417676162 | 1.66 to <2.18 |
| Barbados | 0.514898613 | 0.72978286 | 0.372277232 | <0.52 |
| Japan | 1.135015539 | 1.250789766 | 1.011147255 | 1.12 to <1.66 |
| American Samoa | 0.784004539 | 1.586849206 | 0.387436607 | 0.77 to <1.12 |
| Palestine | 1.858937316 | 2.860359993 | 1.219521159 | 1.66 to <2.18 |
| Saint Kitts and Nevis | 0.807817422 | 1.08434722 | 0.602002675 | 0.77 to <1.12 |
| Liberia | 7.000388953 | 11.73455755 | 3.597105204 | 5.12 to <22.27 |
| Puerto Rico | 0.423604504 | 0.546995727 | 0.331670854 | <0.52 |
| Qatar | 1.384705555 | 2.162351438 | 0.872606827 | 1.12 to <1.66 |
| Guinea-Bissau | 5.166524762 | 7.805976595 | 2.909777535 | 5.12 to <22.27 |
| Uzbekistan | 1.991362641 | 2.689107051 | 1.438800861 | 1.66 to <2.18 |
| Tonga | 3.626681246 | 7.958307057 | 1.689170641 | 2.86 to <5.12 |
| Nicaragua | 1.824778553 | 2.754219141 | 1.192714049 | 1.66 to <2.18 |
| Finland | 0.470222932 | 0.596371019 | 0.36557237 | <0.52 |
| Portugal | 0.882375032 | 1.1303701 | 0.681377678 | 0.77 to <1.12 |
| Guam | 1.450368457 | 2.094094778 | 0.996508449 | 1.12 to <1.66 |
| Taiwan (Province of China) | 1.790481245 | 2.139770311 | 1.489081968 | 1.66 to <2.18 |
| Serbia | 0.545131249 | 0.982662366 | 0.276238689 | 0.52 to <0.77 |
| Algeria | 1.695986953 | 2.693101172 | 1.039590895 | 1.66 to <2.18 |
| Canada | 1.672589474 | 2.038382044 | 1.359219377 | 1.66 to <2.18 |
| Viet Nam | 4.858086922 | 7.559151453 | 3.012495987 | 2.86 to <5.12 |
| Maldives | 1.093111729 | 1.725425797 | 0.665069288 | 0.77 to <1.12 |
| Ethiopia | 2.83533421 | 5.847667871 | 1.453318168 | 2.18 to <2.86 |
| Senegal | 3.205548741 | 4.654596977 | 2.132685343 | 2.86 to <5.12 |
| Guyana | 0.767192173 | 0.9969991 | 0.561715175 | 0.52 to <0.77 |
| Cyprus | 0.45327041 | 0.711375699 | 0.27975345 | <0.52 |
| Netherlands | 0.479604249 | 0.569160973 | 0.403310015 | <0.52 |
| Albania | 4.868572 | 8.092626524 | 2.934712545 | 2.86 to <5.12 |
| Turkey | 0.939951094 | 1.455174719 | 0.639801234 | 0.77 to <1.12 |
| Romania | 0.552502577 | 0.738472921 | 0.400438789 | 0.52 to <0.77 |
| Bangladesh | 2.269516616 | 3.193750316 | 1.492845604 | 2.18 to <2.86 |
| Gabon | 3.318486698 | 5.886825227 | 1.523701812 | 2.86 to <5.12 |
| Slovakia | 0.71692779 | 1.283626797 | 0.406040756 | 0.52 to <0.77 |
| Brunei Darussalam | 1.562838382 | 2.411982412 | 1.017281527 | 1.12 to <1.66 |
| Rwanda | 3.557158349 | 6.727743593 | 1.922174478 | 2.86 to <5.12 |
| Georgia | 1.887306533 | 2.137480611 | 1.669709647 | 1.66 to <2.18 |
| Zimbabwe | 5.790000732 | 9.216106299 | 3.208417775 | 5.12 to <22.27 |
| Slovenia | 0.291884279 | 0.386794242 | 0.215379569 | <0.52 |
| Malta | 0.71355751 | 0.917694534 | 0.52408629 | 0.52 to <0.77 |
| Belgium | 0.788819749 | 1.033447206 | 0.597123786 | 0.77 to <1.12 |
| Eswatini | 3.987472978 | 9.746637414 | 1.542917711 | 2.86 to <5.12 |
| Grenada | 0.867025643 | 1.107206577 | 0.65917705 | 0.77 to <1.12 |
| San Marino | 0.645818397 | 1.149028948 | 0.37041755 | 0.52 to <0.77 |
| Vanuatu | 0.519399969 | 1.005792467 | 0.232576065 | 0.52 to <0.77 |
| Mozambique | 3.255014645 | 8.545763928 | 1.321697284 | 2.86 to <5.12 |
| New Zealand | 1.44424639 | 1.949380122 | 1.107544261 | 1.12 to <1.66 |
| South Sudan | 11.2831822 | 18.78068625 | 4.631443596 | 5.12 to <22.27 |
| China | 2.563537397 | 3.634022358 | 1.834679286 | 2.18 to <2.86 |
| Saint Lucia | 0.574239639 | 0.781720894 | 0.426758577 | 0.52 to <0.77 |
| Guatemala | 3.411224913 | 4.378784272 | 2.627982863 | 2.86 to <5.12 |
| Morocco | 0.383953573 | 0.683246406 | 0.205547432 | <0.52 |
| Sweden | 0.780407316 | 0.993579717 | 0.612522805 | 0.77 to <1.12 |
| El Salvador | 0.773613252 | 1.195415093 | 0.52784622 | 0.77 to <1.12 |
| Djibouti | 3.694816007 | 7.521994465 | 1.710733458 | 2.86 to <5.12 |
| Azerbaijan | 3.66577587 | 6.527318432 | 1.994846637 | 2.86 to <5.12 |
| Jamaica | 0.546875725 | 0.740583181 | 0.392228797 | 0.52 to <0.77 |
| Cambodia | 2.521399391 | 4.770116197 | 1.318755416 | 2.18 to <2.86 |
| Lesotho | 2.616004382 | 7.389845752 | 1.011648668 | 2.18 to <2.86 |
| Denmark | 0.286292233 | 0.356107034 | 0.219783643 | <0.52 |
| Norway | 1.818968819 | 2.179060995 | 1.520870668 | 1.66 to <2.18 |
| Nigeria | 3.600623993 | 4.89811412 | 2.329405447 | 2.86 to <5.12 |
| Tuvalu | 0.706267443 | 1.363170157 | 0.385394679 | 0.52 to <0.77 |
| Egypt | 5.015757511 | 6.961665577 | 3.722743464 | 2.86 to <5.12 |
| Lao People's Democratic Republic | 3.296159563 | 5.004704913 | 2.005263888 | 2.86 to <5.12 |
| Solomon Islands | 0.792765068 | 1.867252762 | 0.383702792 | 0.77 to <1.12 |
| Philippines | 2.903310698 | 3.776577119 | 2.260880738 | 2.86 to <5.12 |
| Fiji | 1.224560644 | 2.091474063 | 0.656613366 | 1.12 to <1.66 |
| Togo | 2.51288809 | 4.210358123 | 1.450382062 | 2.18 to <2.86 |
| Cook Islands | 0.402638899 | 0.89371706 | 0.197700659 | <0.52 |
| Bahamas | 0.755415892 | 1.052619934 | 0.548718228 | 0.52 to <0.77 |
| Switzerland | 0.651212463 | 0.800471144 | 0.518216508 | 0.52 to <0.77 |
| Comoros | 4.461379649 | 7.669313212 | 2.565910806 | 2.86 to <5.12 |
| United Republic of Tanzania | 5.959307641 | 10.16205169 | 3.391090955 | 5.12 to <22.27 |
| Marshall Islands | 0.511605983 | 1.175175115 | 0.207928512 | <0.52 |
| Gambia | 17.24215577 | 25.75050795 | 11.17802775 | 5.12 to <22.27 |
| Turkmenistan | 2.025841629 | 2.578764653 | 1.573471935 | 1.66 to <2.18 |
| Greece | 2.003033997 | 2.387662881 | 1.655397638 | 1.66 to <2.18 |
| Suriname | 1.149250584 | 1.752297566 | 0.727685734 | 1.12 to <1.66 |
| India | 2.190903591 | 3.034404726 | 1.581671297 | 2.18 to <2.86 |
| Burundi | 2.355903321 | 5.307542367 | 0.947418689 | 2.18 to <2.86 |
| Mexico | 2.590402282 | 3.45950984 | 1.94254121 | 2.18 to <2.86 |
| Mauritania | 6.753089908 | 10.96131933 | 3.314694281 | 5.12 to <22.27 |
| Burkina Faso | 12.46177525 | 21.31084776 | 6.118352827 | 5.12 to <22.27 |
| Hungary | 0.302569329 | 0.393180886 | 0.222805191 | <0.52 |
| Syrian Arab Republic | 2.443793748 | 3.755261362 | 1.196678653 | 2.18 to <2.86 |
| Central African Republic | 5.298606709 | 9.525248594 | 2.578375026 | 5.12 to <22.27 |
| Lithuania | 1.788760662 | 2.312102177 | 1.384227476 | 1.66 to <2.18 |
| Pakistan | 7.127753342 | 9.965391807 | 4.88102528 | 5.12 to <22.27 |
| Paraguay | 2.470717106 | 4.030772511 | 1.479700142 | 2.18 to <2.86 |
| Cameroon | 5.614718111 | 11.07209042 | 2.153169296 | 5.12 to <22.27 |
| Croatia | 0.267122696 | 0.396970142 | 0.18390689 | <0.52 |
| Sudan | 4.851937711 | 7.731159575 | 3.037817281 | 2.86 to <5.12 |
| Tunisia | 1.050697956 | 1.638043976 | 0.667134561 | 0.77 to <1.12 |
| Ireland | 0.481663178 | 0.585520036 | 0.376449547 | <0.52 |
| Uganda | 9.777968357 | 17.85870897 | 5.262017578 | 5.12 to <22.27 |
| Antigua and Barbuda | 0.662722603 | 0.795806916 | 0.547808511 | 0.52 to <0.77 |
| Honduras | 1.215620178 | 2.047789661 | 0.695647589 | 1.12 to <1.66 |
| Niue | 1.946379889 | 4.160534506 | 0.964906233 | 1.66 to <2.18 |
| Madagascar | 2.400423105 | 4.692350214 | 1.196632392 | 2.18 to <2.86 |
| Saint Vincent and the Grenadines | 0.762594006 | 0.97859063 | 0.586567536 | 0.52 to <0.77 |
| Estonia | 2.302048878 | 3.00195715 | 1.711734083 | 2.18 to <2.86 |
| Chile | 0.327452867 | 0.419678729 | 0.251779056 | <0.52 |
| Malawi | 8.801358788 | 20.32862488 | 3.825188349 | 5.12 to <22.27 |
| Kazakhstan | 1.295516394 | 1.642434828 | 0.999983465 | 1.12 to <1.66 |
| Somalia | 5.600016222 | 11.31147388 | 2.192025284 | 5.12 to <22.27 |
| Benin | 8.754861827 | 14.22824135 | 4.912901192 | 5.12 to <22.27 |
| Papua New Guinea | 1.30232104 | 2.397649702 | 0.688971713 | 1.12 to <1.66 |
| Monaco | 1.112851079 | 1.715373838 | 0.726809853 | 0.77 to <1.12 |
| Bermuda | 0.294986707 | 0.394527374 | 0.21029091 | <0.52 |
| Saudi Arabia | 0.914702153 | 1.569073446 | 0.556757841 | 0.77 to <1.12 |
| Andorra | 3.503390972 | 5.597043486 | 2.036871122 | 2.86 to <5.12 |
| Congo | 2.916007647 | 6.375536235 | 1.123778432 | 2.86 to <5.12 |
| Iran (Islamic Republic of) | 2.053829038 | 3.376064689 | 1.410154173 | 1.66 to <2.18 |
| Italy | 0.779026863 | 0.930579323 | 0.632726876 | 0.77 to <1.12 |
| Palau | 1.280050689 | 2.666960995 | 0.596486924 | 1.12 to <1.66 |
| Namibia | 2.931613497 | 4.545625493 | 1.831651849 | 2.86 to <5.12 |
| Bahrain | 0.913214063 | 1.265069953 | 0.597843011 | 0.77 to <1.12 |
| Nepal | 2.313486602 | 3.439775693 | 1.407806984 | 2.18 to <2.86 |
| Thailand | 2.86269146 | 3.839950311 | 2.083381349 | 2.18 to <2.86 |
| Lebanon | 0.805821259 | 1.607955579 | 0.420005387 | 0.77 to <1.12 |
| Dominica | 1.530982025 | 2.382371858 | 0.989039269 | 1.12 to <1.66 |
| Sri Lanka | 0.283105617 | 0.461661128 | 0.180710144 | <0.52 |
| Ecuador | 1.172442532 | 1.595678727 | 0.853527481 | 1.12 to <1.66 |
| Mongolia | 15.83092985 | 23.88197924 | 10.00475581 | 5.12 to <22.27 |
| Spain | 1.338204328 | 1.625354191 | 1.104434102 | 1.12 to <1.66 |
| Cuba | 0.402717242 | 0.52266655 | 0.300173595 | <0.52 |
| Russian Federation | 2.212559997 | 2.43293678 | 1.98065254 | 2.18 to <2.86 |
| Montenegro | 0.942345577 | 1.521018618 | 0.518849401 | 0.77 to <1.12 |
| Tokelau | 0.756173169 | 2.261652724 | 0.289147156 | 0.52 to <0.77 |
| Niger | 5.059154541 | 8.25539938 | 3.023015447 | 2.86 to <5.12 |
| Ghana | 2.520844712 | 4.490257184 | 1.442811862 | 2.18 to <2.86 |
| Northern Mariana Islands | 0.341278346 | 0.633366382 | 0.184238277 | <0.52 |
| Equatorial Guinea | 2.563072366 | 4.788795049 | 1.284821416 | 2.18 to <2.86 |
| Afghanistan | 5.846783561 | 8.84780082 | 3.803820564 | 5.12 to <22.27 |
| Republic of Korea | 1.335072749 | 2.153853133 | 0.832169928 | 1.12 to <1.66 |
| Yemen | 2.225060399 | 3.740221008 | 1.217130643 | 2.18 to <2.86 |
| Bosnia and Herzegovina | 0.70749434 | 0.971459179 | 0.494598906 | 0.52 to <0.77 |
| France | 1.659994714 | 2.021962738 | 1.333481745 | 1.66 to <2.18 |
| Indonesia | 1.852371025 | 3.233164124 | 0.842641645 | 1.66 to <2.18 |
| Venezuela (Bolivarian Republic of) | 3.231457771 | 4.396167557 | 2.334218734 | 2.86 to <5.12 |
| Bolivia (Plurinational State of) | 2.626843149 | 4.286285769 | 1.665594873 | 2.18 to <2.86 |
| Argentina | 0.460622048 | 0.565974089 | 0.368922301 | <0.52 |
| Zambia | 6.98026755 | 15.47678256 | 3.102153275 | 5.12 to <22.27 |
| Eritrea | 3.707725438 | 8.447871556 | 1.582048993 | 2.86 to <5.12 |
| Cabo Verde | 1.997660626 | 3.182176003 | 1.238533792 | 1.66 to <2.18 |
| Sao Tome and Principe | 0.834399447 | 2.022697689 | 0.346695228 | 0.77 to <1.12 |
| United States Virgin Islands | 0.292646007 | 0.583350024 | 0.142160594 | <0.52 |
| Israel | 0.540958253 | 0.668409766 | 0.425996016 | 0.52 to <0.77 |
| Dominican Republic | 1.083952257 | 1.556259698 | 0.699251891 | 0.77 to <1.12 |
| Kuwait | 0.323901089 | 0.416858289 | 0.248968838 | <0.52 |
| Malaysia | 1.208494683 | 1.744817366 | 0.783897054 | 1.12 to <1.66 |
| Peru | 1.078566632 | 1.871551328 | 0.575111874 | 0.77 to <1.12 |
| Brazil | 1.27545794 | 1.574607035 | 0.984079161 | 1.12 to <1.66 |
| South Africa | 1.817057217 | 2.379329448 | 1.317224452 | 1.66 to <2.18 |
| Guinea | 16.22248639 | 24.95157885 | 9.122312644 | 5.12 to <22.27 |
| Republic of Moldova | 6.13892494 | 8.195991671 | 4.697183274 | 5.12 to <22.27 |
| Kiribati | 1.53026768 | 3.447547005 | 0.688578926 | 1.12 to <1.66 |
| Kyrgyzstan | 0.667524453 | 0.881437849 | 0.499443372 | 0.52 to <0.77 |
| North Macedonia | 1.021962029 | 1.560708943 | 0.662031022 | 0.77 to <1.12 |
| Democratic Republic of the Congo | 1.725155259 | 3.696637225 | 0.863520949 | 1.66 to <2.18 |
| Greenland | 2.552408662 | 4.055698944 | 1.53050509 | 2.18 to <2.86 |
| Uruguay | 0.26964107 | 0.361144713 | 0.193024421 | <0.52 |
| Libya | 7.647724769 | 11.44658552 | 4.25841309 | 5.12 to <22.27 |
| Mauritius | 0.11955173 | 0.137647647 | 0.099300656 | <0.52 |

**Supplementary Table S5**: Prediction of hepatoblastoma age-standardized rates by sex, 2021-2030

| year | sex | measure | pred_val | pred_low | pred_up |
| --- | --- | --- | --- | --- | --- |
| 1990 | Male | Deaths | 0.545353766 | 0.52576326 | 0.564944271 |
| 1991 | Male | Deaths | 0.51751945 | 0.501082949 | 0.533955951 |
| 1992 | Male | Deaths | 0.489915442 | 0.474622504 | 0.505208381 |
| 1993 | Male | Deaths | 0.462543501 | 0.448122268 | 0.476964735 |
| 1994 | Male | Deaths | 0.435827637 | 0.42223229 | 0.449422983 |
| 1995 | Male | Deaths | 0.408211625 | 0.395449792 | 0.420973457 |
| 1996 | Male | Deaths | 0.380580505 | 0.368636762 | 0.392524248 |
| 1997 | Male | Deaths | 0.352398079 | 0.341264965 | 0.363531193 |
| 1998 | Male | Deaths | 0.323904337 | 0.313576242 | 0.334232431 |
| 1999 | Male | Deaths | 0.296924696 | 0.287360832 | 0.306488559 |
| 2000 | Male | Deaths | 0.27319629 | 0.264295419 | 0.282097161 |
| 2001 | Male | Deaths | 0.250797804 | 0.242501657 | 0.259093952 |
| 2002 | Male | Deaths | 0.228103587 | 0.220415619 | 0.235791555 |
| 2003 | Male | Deaths | 0.206822255 | 0.199692111 | 0.213952398 |
| 2004 | Male | Deaths | 0.190023255 | 0.183348679 | 0.196697832 |
| 2005 | Male | Deaths | 0.177092488 | 0.170794999 | 0.183389977 |
| 2006 | Male | Deaths | 0.166811679 | 0.160828361 | 0.172794997 |
| 2007 | Male | Deaths | 0.157789293 | 0.152084959 | 0.163493626 |
| 2008 | Male | Deaths | 0.149999165 | 0.144538343 | 0.155459986 |
| 2009 | Male | Deaths | 0.141257659 | 0.136083678 | 0.14643164 |
| 2010 | Male | Deaths | 0.131252934 | 0.126435437 | 0.136070431 |
| 2011 | Male | Deaths | 0.121526189 | 0.117054489 | 0.12599789 |
| 2012 | Male | Deaths | 0.113047781 | 0.108880722 | 0.117214841 |
| 2013 | Male | Deaths | 0.106042467 | 0.102131308 | 0.109953625 |
| 2014 | Male | Deaths | 0.099037861 | 0.095369689 | 0.102706032 |
| 2015 | Male | Deaths | 0.092643688 | 0.089194129 | 0.096093248 |
| 2016 | Male | Deaths | 0.085859653 | 0.082633786 | 0.08908552 |
| 2017 | Male | Deaths | 0.078488638 | 0.075504764 | 0.081472513 |
| 2018 | Male | Deaths | 0.071170523 | 0.068425165 | 0.07391588 |
| 2019 | Male | Deaths | 0.064380115 | 0.061854104 | 0.066906125 |
| 2020 | Male | Deaths | 0.058530101 | 0.05613567 | 0.060924532 |
| 2021 | Male | Deaths | 0.053393237 | 0.050787042 | 0.055999432 |
| 2022 | Male | Deaths | 0.048446541 | 0.043332759 | 0.053560323 |
| 2023 | Male | Deaths | 0.043970182 | 0.037000689 | 0.050939676 |
| 2024 | Male | Deaths | 0.039891808 | 0.030958953 | 0.048824663 |
| 2025 | Male | Deaths | 0.036192677 | 0.025347427 | 0.047037928 |
| 2026 | Male | Deaths | 0.032841501 | 0.020200218 | 0.045482784 |
| 2027 | Male | Deaths | 0.029802845 | 0.01551727 | 0.044088419 |
| 2028 | Male | Deaths | 0.027047386 | 0.011311054 | 0.042783718 |
| 2029 | Male | Deaths | 0.024548555 | 0.007571528 | 0.041525583 |
| 2030 | Male | Deaths | 0.022282283 | 0.004276735 | 0.040287832 |
| 1990 | Female | Deaths | 0.193057994 | 0.18600398 | 0.200112007 |
| 1991 | Female | Deaths | 0.180931195 | 0.174944347 | 0.186918043 |
| 1992 | Female | Deaths | 0.17004338 | 0.164582324 | 0.175504436 |
| 1993 | Female | Deaths | 0.158613836 | 0.153533291 | 0.163694382 |
| 1994 | Female | Deaths | 0.147901827 | 0.143138821 | 0.152664834 |
| 1995 | Female | Deaths | 0.137801072 | 0.13332793 | 0.142274214 |
| 1996 | Female | Deaths | 0.127577355 | 0.123394981 | 0.131759729 |
| 1997 | Female | Deaths | 0.117282421 | 0.113402289 | 0.121162553 |
| 1998 | Female | Deaths | 0.107477425 | 0.10388339 | 0.11107146 |
| 1999 | Female | Deaths | 0.098715578 | 0.095379179 | 0.102051976 |
| 2000 | Female | Deaths | 0.091021897 | 0.087909489 | 0.094134305 |
| 2001 | Female | Deaths | 0.084011193 | 0.081103297 | 0.086919089 |
| 2002 | Female | Deaths | 0.077817028 | 0.07508547 | 0.080548586 |
| 2003 | Female | Deaths | 0.072174704 | 0.0696055 | 0.074743909 |
| 2004 | Female | Deaths | 0.067754337 | 0.06531517 | 0.070193504 |
| 2005 | Female | Deaths | 0.064386819 | 0.0620518 | 0.066721838 |
| 2006 | Female | Deaths | 0.061843093 | 0.059585668 | 0.064100518 |
| 2007 | Female | Deaths | 0.059656382 | 0.05745898 | 0.061853783 |
| 2008 | Female | Deaths | 0.057827034 | 0.055673816 | 0.059980251 |
| 2009 | Female | Deaths | 0.055293338 | 0.053223649 | 0.057363027 |
| 2010 | Female | Deaths | 0.051980224 | 0.050045616 | 0.053914831 |
| 2011 | Female | Deaths | 0.04857374 | 0.046774831 | 0.050372648 |
| 2012 | Female | Deaths | 0.045473065 | 0.043791186 | 0.047154944 |
| 2013 | Female | Deaths | 0.042819752 | 0.041237175 | 0.04440233 |
| 2014 | Female | Deaths | 0.040240006 | 0.038748939 | 0.041731074 |
| 2015 | Female | Deaths | 0.037834846 | 0.036427744 | 0.039241948 |
| 2016 | Female | Deaths | 0.035567277 | 0.034235802 | 0.036898752 |
| 2017 | Female | Deaths | 0.033146953 | 0.031893053 | 0.034400852 |
| 2018 | Female | Deaths | 0.030770602 | 0.029592637 | 0.031948566 |
| 2019 | Female | Deaths | 0.028571675 | 0.027459745 | 0.029683604 |
| 2020 | Female | Deaths | 0.026438707 | 0.02535645 | 0.027520963 |
| 2021 | Female | Deaths | 0.024468259 | 0.023308411 | 0.025628107 |
| 2022 | Female | Deaths | 0.022712443 | 0.020602168 | 0.024822718 |
| 2023 | Female | Deaths | 0.021038022 | 0.018346608 | 0.023729435 |
| 2024 | Female | Deaths | 0.01948405 | 0.016143347 | 0.022824753 |
| 2025 | Female | Deaths | 0.018042787 | 0.014036113 | 0.02204946 |
| 2026 | Female | Deaths | 0.016707612 | 0.012045252 | 0.021369973 |
| 2027 | Female | Deaths | 0.015471859 | 0.010181093 | 0.020762626 |
| 2028 | Female | Deaths | 0.014328082 | 0.00845258 | 0.020203584 |
| 2029 | Female | Deaths | 0.013269395 | 0.006861773 | 0.019677017 |
| 2030 | Female | Deaths | 0.012289427 | 0.00540673 | 0.019172125 |
| 1990 | Both | Deaths | 0.29277206 | 0.271548731 | 0.313995389 |
| 1991 | Both | Deaths | 0.275838711 | 0.256568887 | 0.295108536 |
| 1992 | Both | Deaths | 0.260262579 | 0.242168772 | 0.278356386 |
| 1993 | Both | Deaths | 0.244480437 | 0.227393021 | 0.261567854 |
| 1994 | Both | Deaths | 0.229506195 | 0.213352718 | 0.245659673 |
| 1995 | Both | Deaths | 0.214868318 | 0.199641479 | 0.230095158 |
| 1996 | Both | Deaths | 0.20004598 | 0.185765743 | 0.214326217 |
| 1997 | Both | Deaths | 0.184902824 | 0.171576526 | 0.198229123 |
| 1998 | Both | Deaths | 0.169937937 | 0.15756147 | 0.182314405 |
| 1999 | Both | Deaths | 0.156130936 | 0.144648286 | 0.167613586 |
| 2000 | Both | Deaths | 0.14400789 | 0.133321227 | 0.154694552 |
| 2001 | Both | Deaths | 0.132561265 | 0.122617372 | 0.142505157 |
| 2002 | Both | Deaths | 0.1216595 | 0.112416424 | 0.130902577 |
| 2003 | Both | Deaths | 0.111458225 | 0.102854175 | 0.120062275 |
| 2004 | Both | Deaths | 0.103423369 | 0.095340081 | 0.111506658 |
| 2005 | Both | Deaths | 0.097356513 | 0.089692694 | 0.105020332 |
| 2006 | Both | Deaths | 0.092774754 | 0.085443962 | 0.100105546 |
| 2007 | Both | Deaths | 0.088837058 | 0.081790322 | 0.095883794 |
| 2008 | Both | Deaths | 0.08548819 | 0.078686945 | 0.092289434 |
| 2009 | Both | Deaths | 0.081239369 | 0.074747479 | 0.08773126 |
| 2010 | Both | Deaths | 0.075972952 | 0.069877918 | 0.082067987 |
| 2011 | Both | Deaths | 0.070680658 | 0.064973845 | 0.076387472 |
| 2012 | Both | Deaths | 0.066000665 | 0.060638073 | 0.071363257 |
| 2013 | Both | Deaths | 0.06204789 | 0.056982547 | 0.067113232 |
| 2014 | Both | Deaths | 0.058128834 | 0.05334901 | 0.062908659 |
| 2015 | Both | Deaths | 0.0545295 | 0.05002429 | 0.05903471 |
| 2016 | Both | Deaths | 0.0509501 | 0.046712367 | 0.055187833 |
| 2017 | Both | Deaths | 0.047129691 | 0.043164378 | 0.051095003 |
| 2018 | Both | Deaths | 0.043342252 | 0.039640333 | 0.047044171 |
| 2019 | Both | Deaths | 0.039800948 | 0.036339651 | 0.043262244 |
| 2020 | Both | Deaths | 0.036548627 | 0.033270095 | 0.039827159 |
| 2021 | Both | Deaths | 0.033575876 | 0.030342412 | 0.03680934 |
| 2022 | Both | Deaths | 0.031293066 | 0.026939892 | 0.035646241 |
| 2023 | Both | Deaths | 0.02879785 | 0.023605091 | 0.033990609 |
| 2024 | Both | Deaths | 0.026549652 | 0.02036728 | 0.032732025 |
| 2025 | Both | Deaths | 0.024503182 | 0.017271335 | 0.031735029 |
| 2026 | Both | Deaths | 0.022641125 | 0.014342956 | 0.030939294 |
| 2027 | Both | Deaths | 0.020953999 | 0.011587573 | 0.030320424 |
| 2028 | Both | Deaths | 0.019448595 | 0.009010736 | 0.029886454 |
| 2029 | Both | Deaths | 0.018115116 | 0.00659017 | 0.029640061 |
| 2030 | Both | Deaths | 0.016930497 | 0.004291773 | 0.029569222 |
| 1990 | Male | DALYs (Disability-Adjusted Life Years) | 47.92106417 | 47.72021439 | 48.12191396 |
| 1991 | Male | DALYs (Disability-Adjusted Life Years) | 45.23547907 | 45.04635352 | 45.42460461 |
| 1992 | Male | DALYs (Disability-Adjusted Life Years) | 42.77570695 | 42.59627768 | 42.95513623 |
| 1993 | Male | DALYs (Disability-Adjusted Life Years) | 40.4323336 | 40.26237073 | 40.60229648 |
| 1994 | Male | DALYs (Disability-Adjusted Life Years) | 38.19804672 | 38.03719745 | 38.35889599 |
| 1995 | Male | DALYs (Disability-Adjusted Life Years) | 35.73903918 | 35.58804224 | 35.89003611 |
| 1996 | Male | DALYs (Disability-Adjusted Life Years) | 33.38294614 | 33.24135595 | 33.52453632 |
| 1997 | Male | DALYs (Disability-Adjusted Life Years) | 31.03623106 | 30.90361752 | 31.16884461 |
| 1998 | Male | DALYs (Disability-Adjusted Life Years) | 28.49030796 | 28.36709058 | 28.61352535 |
| 1999 | Male | DALYs (Disability-Adjusted Life Years) | 26.01555725 | 25.90145504 | 26.12965947 |
| 2000 | Male | DALYs (Disability-Adjusted Life Years) | 24.05368535 | 23.94713946 | 24.16023124 |
| 2001 | Male | DALYs (Disability-Adjusted Life Years) | 22.26272887 | 22.16277452 | 22.36268323 |
| 2002 | Male | DALYs (Disability-Adjusted Life Years) | 20.12463002 | 20.03215582 | 20.21710423 |
| 2003 | Male | DALYs (Disability-Adjusted Life Years) | 18.03828666 | 17.95272904 | 18.12384429 |
| 2004 | Male | DALYs (Disability-Adjusted Life Years) | 16.58201736 | 16.50167947 | 16.66235524 |
| 2005 | Male | DALYs (Disability-Adjusted Life Years) | 15.52415566 | 15.44790295 | 15.60040837 |
| 2006 | Male | DALYs (Disability-Adjusted Life Years) | 14.65500317 | 14.58227158 | 14.72773475 |
| 2007 | Male | DALYs (Disability-Adjusted Life Years) | 13.87504925 | 13.80551875 | 13.94457975 |
| 2008 | Male | DALYs (Disability-Adjusted Life Years) | 13.31322612 | 13.24633622 | 13.38011603 |
| 2009 | Male | DALYs (Disability-Adjusted Life Years) | 12.56849381 | 12.5049409 | 12.63204672 |
| 2010 | Male | DALYs (Disability-Adjusted Life Years) | 11.58151996 | 11.52237973 | 11.64066019 |
| 2011 | Male | DALYs (Disability-Adjusted Life Years) | 10.65977417 | 10.60485344 | 10.71469489 |
| 2012 | Male | DALYs (Disability-Adjusted Life Years) | 9.912971955 | 9.861700336 | 9.964243574 |
| 2013 | Male | DALYs (Disability-Adjusted Life Years) | 9.352500673 | 9.30414274 | 9.400858606 |
| 2014 | Male | DALYs (Disability-Adjusted Life Years) | 8.731851631 | 8.686351862 | 8.777351399 |
| 2015 | Male | DALYs (Disability-Adjusted Life Years) | 8.217964293 | 8.17498927 | 8.260939316 |
| 2016 | Male | DALYs (Disability-Adjusted Life Years) | 7.630263655 | 7.589996551 | 7.670530759 |
| 2017 | Male | DALYs (Disability-Adjusted Life Years) | 6.947408375 | 6.910127313 | 6.984689436 |
| 2018 | Male | DALYs (Disability-Adjusted Life Years) | 6.269170029 | 6.234818302 | 6.303521756 |
| 2019 | Male | DALYs (Disability-Adjusted Life Years) | 5.646509155 | 5.614843278 | 5.678175031 |
| 2020 | Male | DALYs (Disability-Adjusted Life Years) | 5.133578252 | 5.104064005 | 5.163092498 |
| 2021 | Male | DALYs (Disability-Adjusted Life Years) | 4.723321011 | 4.695156049 | 4.751485973 |
| 2022 | Male | DALYs (Disability-Adjusted Life Years) | 4.306717606 | 3.995469167 | 4.617966045 |
| 2023 | Male | DALYs (Disability-Adjusted Life Years) | 3.93029668 | 3.450477815 | 4.410115545 |
| 2024 | Male | DALYs (Disability-Adjusted Life Years) | 3.584113745 | 2.922951411 | 4.245276079 |
| 2025 | Male | DALYs (Disability-Adjusted Life Years) | 3.268141705 | 2.428844544 | 4.107438865 |
| 2026 | Male | DALYs (Disability-Adjusted Life Years) | 2.980494786 | 1.971791256 | 3.989198316 |
| 2027 | Male | DALYs (Disability-Adjusted Life Years) | 2.718347347 | 1.550955349 | 3.885739345 |
| 2028 | Male | DALYs (Disability-Adjusted Life Years) | 2.479424344 | 1.168628742 | 3.790219946 |
| 2029 | Male | DALYs (Disability-Adjusted Life Years) | 2.261654049 | 0.824826678 | 3.69848142 |
| 2030 | Male | DALYs (Disability-Adjusted Life Years) | 2.063150125 | 0.518347844 | 3.607952407 |
| 1990 | Female | DALYs (Disability-Adjusted Life Years) | 17.00128055 | 16.92711554 | 17.07544557 |
| 1991 | Female | DALYs (Disability-Adjusted Life Years) | 15.85880418 | 15.7897425 | 15.92786585 |
| 1992 | Female | DALYs (Disability-Adjusted Life Years) | 14.94571793 | 14.88039935 | 15.01103651 |
| 1993 | Female | DALYs (Disability-Adjusted Life Years) | 13.95090909 | 13.88950094 | 14.01231723 |
| 1994 | Female | DALYs (Disability-Adjusted Life Years) | 13.04387206 | 12.98605797 | 13.10168616 |
| 1995 | Female | DALYs (Disability-Adjusted Life Years) | 12.21277934 | 12.15827135 | 12.26728733 |
| 1996 | Female | DALYs (Disability-Adjusted Life Years) | 11.32961409 | 11.27845976 | 11.38076843 |
| 1997 | Female | DALYs (Disability-Adjusted Life Years) | 10.40491253 | 10.35718869 | 10.45263637 |
| 1998 | Female | DALYs (Disability-Adjusted Life Years) | 9.499729801 | 9.455304373 | 9.544155229 |
| 1999 | Female | DALYs (Disability-Adjusted Life Years) | 8.71856231 | 8.677071601 | 8.76005302 |
| 2000 | Female | DALYs (Disability-Adjusted Life Years) | 8.058228482 | 8.019278882 | 8.097178082 |
| 2001 | Female | DALYs (Disability-Adjusted Life Years) | 7.442835591 | 7.406276872 | 7.479394311 |
| 2002 | Female | DALYs (Disability-Adjusted Life Years) | 6.868844012 | 6.834507977 | 6.903180047 |
| 2003 | Female | DALYs (Disability-Adjusted Life Years) | 6.316986309 | 6.284751058 | 6.34922156 |
| 2004 | Female | DALYs (Disability-Adjusted Life Years) | 5.917777876 | 5.887187885 | 5.948367868 |
| 2005 | Female | DALYs (Disability-Adjusted Life Years) | 5.643350053 | 5.613963073 | 5.672737033 |
| 2006 | Female | DALYs (Disability-Adjusted Life Years) | 5.441247617 | 5.412732808 | 5.469762427 |
| 2007 | Female | DALYs (Disability-Adjusted Life Years) | 5.265851554 | 5.238075567 | 5.293627541 |
| 2008 | Female | DALYs (Disability-Adjusted Life Years) | 5.170820043 | 5.143577439 | 5.198062648 |
| 2009 | Female | DALYs (Disability-Adjusted Life Years) | 4.954435502 | 4.928234083 | 4.980636922 |
| 2010 | Female | DALYs (Disability-Adjusted Life Years) | 4.61189638 | 4.587350764 | 4.636441996 |
| 2011 | Female | DALYs (Disability-Adjusted Life Years) | 4.272906588 | 4.250044225 | 4.295768952 |
| 2012 | Female | DALYs (Disability-Adjusted Life Years) | 3.986310743 | 3.964924027 | 4.00769746 |
| 2013 | Female | DALYs (Disability-Adjusted Life Years) | 3.775975972 | 3.755766427 | 3.796185518 |
| 2014 | Female | DALYs (Disability-Adjusted Life Years) | 3.554776231 | 3.535683391 | 3.573869072 |
| 2015 | Female | DALYs (Disability-Adjusted Life Years) | 3.351580972 | 3.333511885 | 3.369650059 |
| 2016 | Female | DALYs (Disability-Adjusted Life Years) | 3.170197666 | 3.153039852 | 3.18735548 |
| 2017 | Female | DALYs (Disability-Adjusted Life Years) | 2.946141858 | 2.929983484 | 2.962300233 |
| 2018 | Female | DALYs (Disability-Adjusted Life Years) | 2.725451619 | 2.710264101 | 2.740639138 |
| 2019 | Female | DALYs (Disability-Adjusted Life Years) | 2.526770031 | 2.512475449 | 2.541064612 |
| 2020 | Female | DALYs (Disability-Adjusted Life Years) | 2.329027813 | 2.315550996 | 2.342504629 |
| 2021 | Female | DALYs (Disability-Adjusted Life Years) | 2.156233323 | 2.143335382 | 2.169131264 |
| 2022 | Female | DALYs (Disability-Adjusted Life Years) | 1.995348368 | 1.872928131 | 2.117768605 |
| 2023 | Female | DALYs (Disability-Adjusted Life Years) | 1.844922371 | 1.667797003 | 2.022047738 |
| 2024 | Female | DALYs (Disability-Adjusted Life Years) | 1.705616584 | 1.467029214 | 1.944203954 |
| 2025 | Female | DALYs (Disability-Adjusted Life Years) | 1.57654085 | 1.275305867 | 1.877775832 |
| 2026 | Female | DALYs (Disability-Adjusted Life Years) | 1.457116233 | 1.0942836 | 1.819948866 |
| 2027 | Female | DALYs (Disability-Adjusted Life Years) | 1.346795849 | 0.924506275 | 1.769085424 |
| 2028 | Female | DALYs (Disability-Adjusted Life Years) | 1.244881493 | 0.767020798 | 1.722742188 |
| 2029 | Female | DALYs (Disability-Adjusted Life Years) | 1.150728607 | 0.622122868 | 1.679334346 |
| 2030 | Female | DALYs (Disability-Adjusted Life Years) | 1.063742212 | 0.489687917 | 1.637796508 |
| 1990 | Both | DALYs (Disability-Adjusted Life Years) | 25.76322867 | 25.55244682 | 25.97401051 |
| 1991 | Both | DALYs (Disability-Adjusted Life Years) | 24.15341999 | 23.95486947 | 24.35197051 |
| 1992 | Both | DALYs (Disability-Adjusted Life Years) | 22.80624324 | 22.61743693 | 22.99504955 |
| 1993 | Both | DALYs (Disability-Adjusted Life Years) | 21.44100167 | 21.2619576 | 21.62004575 |
| 1994 | Both | DALYs (Disability-Adjusted Life Years) | 20.18014229 | 20.01050675 | 20.34977784 |
| 1995 | Both | DALYs (Disability-Adjusted Life Years) | 18.92658713 | 18.76653153 | 19.08664273 |
| 1996 | Both | DALYs (Disability-Adjusted Life Years) | 17.6539284 | 17.50362205 | 17.80423476 |
| 1997 | Both | DALYs (Disability-Adjusted Life Years) | 16.34136196 | 16.2006793 | 16.48204461 |
| 1998 | Both | DALYs (Disability-Adjusted Life Years) | 14.98313678 | 14.85246606 | 15.11380749 |
| 1999 | Both | DALYs (Disability-Adjusted Life Years) | 13.73449067 | 13.61324994 | 13.8557314 |
| 2000 | Both | DALYs (Disability-Adjusted Life Years) | 12.71421939 | 12.60099291 | 12.82744588 |
| 2001 | Both | DALYs (Disability-Adjusted Life Years) | 11.75724071 | 11.65136327 | 11.86311816 |
| 2002 | Both | DALYs (Disability-Adjusted Life Years) | 10.73793924 | 10.63989728 | 10.8359812 |
| 2003 | Both | DALYs (Disability-Adjusted Life Years) | 9.740199992 | 9.64945693 | 9.830943053 |
| 2004 | Both | DALYs (Disability-Adjusted Life Years) | 9.030994275 | 8.945640224 | 9.116348326 |
| 2005 | Both | DALYs (Disability-Adjusted Life Years) | 8.535285344 | 8.453982047 | 8.616588641 |
| 2006 | Both | DALYs (Disability-Adjusted Life Years) | 8.158361507 | 8.080413283 | 8.236309731 |
| 2007 | Both | DALYs (Disability-Adjusted Life Years) | 7.827877802 | 7.75286446 | 7.902891143 |
| 2008 | Both | DALYs (Disability-Adjusted Life Years) | 7.615114843 | 7.542286313 | 7.687943374 |
| 2009 | Both | DALYs (Disability-Adjusted Life Years) | 7.25302459 | 7.183404535 | 7.322644644 |
| 2010 | Both | DALYs (Disability-Adjusted Life Years) | 6.722475585 | 6.657350581 | 6.787600588 |
| 2011 | Both | DALYs (Disability-Adjusted Life Years) | 6.209214205 | 6.148314984 | 6.270113425 |
| 2012 | Both | DALYs (Disability-Adjusted Life Years) | 5.787027635 | 5.729759382 | 5.844295888 |
| 2013 | Both | DALYs (Disability-Adjusted Life Years) | 5.472285894 | 5.417974537 | 5.52659725 |
| 2014 | Both | DALYs (Disability-Adjusted Life Years) | 5.130211343 | 5.078907455 | 5.181515232 |
| 2015 | Both | DALYs (Disability-Adjusted Life Years) | 4.834015593 | 4.785531193 | 4.882499993 |
| 2016 | Both | DALYs (Disability-Adjusted Life Years) | 4.535010312 | 4.489318602 | 4.580702022 |
| 2017 | Both | DALYs (Disability-Adjusted Life Years) | 4.181021557 | 4.138329415 | 4.223713699 |
| 2018 | Both | DALYs (Disability-Adjusted Life Years) | 3.829320581 | 3.789530165 | 3.869110997 |
| 2019 | Both | DALYs (Disability-Adjusted Life Years) | 3.505790141 | 3.468678784 | 3.542901498 |
| 2020 | Both | DALYs (Disability-Adjusted Life Years) | 3.21295226 | 3.178141373 | 3.247763147 |
| 2021 | Both | DALYs (Disability-Adjusted Life Years) | 2.964151833 | 2.930993226 | 2.99731044 |
| 2022 | Both | DALYs (Disability-Adjusted Life Years) | 2.764327763 | 2.554337899 | 2.974317626 |
| 2023 | Both | DALYs (Disability-Adjusted Life Years) | 2.547483799 | 2.241585766 | 2.853381832 |
| 2024 | Both | DALYs (Disability-Adjusted Life Years) | 2.35131821 | 1.938195878 | 2.764440541 |
| 2025 | Both | DALYs (Disability-Adjusted Life Years) | 2.172158731 | 1.650268194 | 2.694049269 |
| 2026 | Both | DALYs (Disability-Adjusted Life Years) | 2.008701102 | 1.379885097 | 2.637517106 |
| 2027 | Both | DALYs (Disability-Adjusted Life Years) | 1.860247148 | 1.126584522 | 2.593909773 |
| 2028 | Both | DALYs (Disability-Adjusted Life Years) | 1.727599106 | 0.890553657 | 2.564644556 |
| 2029 | Both | DALYs (Disability-Adjusted Life Years) | 1.610031345 | 0.669888448 | 2.550174242 |
| 2030 | Both | DALYs (Disability-Adjusted Life Years) | 1.505513844 | 0.461741604 | 2.549286084 |

**Supplementary Table S6**: Prediction of hepatoblastoma numbers, 2021-2030

| year | measure | line | pred_val | pred_low | pred_up |
| --- | --- | --- | --- | --- | --- |
| 1990 | Deaths | Observed | 4826.207163 | 4490.744642 | 5161.669683 |
| 1991 | Deaths | Observed | 4827.197368 | 4504.225105 | 5150.16963 |
| 1992 | Deaths | Observed | 4800.565532 | 4481.068696 | 5120.062367 |
| 1993 | Deaths | Observed | 4771.160184 | 4452.647311 | 5089.673057 |
| 1994 | Deaths | Observed | 4734.788239 | 4417.089614 | 5052.486863 |
| 1995 | Deaths | Observed | 4680.896237 | 4364.627658 | 4997.164815 |
| 1996 | Deaths | Observed | 4591.964756 | 4278.554983 | 4905.374529 |
| 1997 | Deaths | Observed | 4478.586362 | 4169.588644 | 4787.584081 |
| 1998 | Deaths | Observed | 4330.198794 | 4027.345413 | 4633.052176 |
| 1999 | Deaths | Observed | 4182.496002 | 3886.146724 | 4478.84528 |
| 2000 | Deaths | Observed | 4044.96771 | 3754.64299 | 4335.29243 |
| 2001 | Deaths | Observed | 3902.058661 | 3617.948826 | 4186.168496 |
| 2002 | Deaths | Observed | 3741.150069 | 3464.240108 | 4018.060031 |
| 2003 | Deaths | Observed | 3575.100214 | 3305.576976 | 3844.623451 |
| 2004 | Deaths | Observed | 3448.691483 | 3184.744507 | 3712.638458 |
| 2005 | Deaths | Observed | 3359.899928 | 3100.083759 | 3619.716098 |
| 2006 | Deaths | Observed | 3289.650989 | 3033.218072 | 3546.083906 |
| 2007 | Deaths | Observed | 3229.044568 | 2975.288375 | 3482.800762 |
| 2008 | Deaths | Observed | 3189.780118 | 2937.358088 | 3442.202149 |
| 2009 | Deaths | Observed | 3150.324087 | 2899.387399 | 3401.260774 |
| 2010 | Deaths | Observed | 3124.305251 | 2874.824937 | 3373.785565 |
| 2011 | Deaths | Observed | 3100.424835 | 2852.184656 | 3348.665015 |
| 2012 | Deaths | Observed | 3085.902169 | 2838.304251 | 3333.500086 |
| 2013 | Deaths | Observed | 3077.488329 | 2830.30917 | 3324.667489 |
| 2014 | Deaths | Observed | 3040.864105 | 2795.319446 | 3286.408765 |
| 2015 | Deaths | Observed | 3005.05827 | 2760.908481 | 3249.20806 |
| 2016 | Deaths | Observed | 2952.597467 | 2710.567955 | 3194.62698 |
| 2017 | Deaths | Observed | 2872.13572 | 2633.534097 | 3110.737344 |
| 2018 | Deaths | Observed | 2777.164671 | 2542.626051 | 3011.703291 |
| 2019 | Deaths | Observed | 2678.724015 | 2448.036574 | 2909.411456 |
| 2020 | Deaths | Observed | 2558.028371 | 2330.287394 | 2785.769348 |
| 2021 | Deaths | Observed | 2416.783401 | 2185.265005 | 2648.301798 |
| 2022 | Deaths | Predicted | 2411.342698 | 2078.134148 | 2744.551248 |
| 2023 | Deaths | Predicted | 2298.170812 | 1884.797671 | 2711.543953 |
| 2024 | Deaths | Predicted | 2218.31736 | 1701.74979 | 2734.88493 |
| 2025 | Deaths | Predicted | 2144.861272 | 1511.521526 | 2778.201018 |
| 2026 | Deaths | Predicted | 2065.856629 | 1308.507147 | 2823.206112 |
| 2027 | Deaths | Predicted | 1977.750745 | 1093.585601 | 2861.915889 |
| 2028 | Deaths | Predicted | 1900.725025 | 880.9637668 | 2920.486284 |
| 2029 | Deaths | Predicted | 1844.425689 | 672.4580779 | 3016.3933 |
| 2030 | Deaths | Predicted | 1798.135724 | 458.6392258 | 3137.632222 |
| 2021 | Deaths | Predicted | 2416.783401 | 2185.265005 | 2648.301798 |
| 1990 | DALYs (Disability-Adjusted Life Years) | Observed | 426325.969 | 422993.9895 | 429657.9484 |
| 1991 | DALYs (Disability-Adjusted Life Years) | Observed | 424319.2084 | 420999.2353 | 427639.1814 |
| 1992 | DALYs (Disability-Adjusted Life Years) | Observed | 422150.1258 | 418828.799 | 425471.4525 |
| 1993 | DALYs (Disability-Adjusted Life Years) | Observed | 419677.061 | 416355.1751 | 422998.9469 |
| 1994 | DALYs (Disability-Adjusted Life Years) | Observed | 417423.1827 | 414103.4295 | 420742.9359 |
| 1995 | DALYs (Disability-Adjusted Life Years) | Observed | 413404.7955 | 410096.2022 | 416713.3888 |
| 1996 | DALYs (Disability-Adjusted Life Years) | Observed | 406276.2336 | 402991.9159 | 409560.5512 |
| 1997 | DALYs (Disability-Adjusted Life Years) | Observed | 396615.4119 | 393368.8517 | 399861.9721 |
| 1998 | DALYs (Disability-Adjusted Life Years) | Observed | 382736.4357 | 379552.3072 | 385920.5642 |
| 1999 | DALYs (Disability-Adjusted Life Years) | Observed | 369063.3148 | 365945.1681 | 372181.4615 |
| 2000 | DALYs (Disability-Adjusted Life Years) | Observed | 358093.5469 | 355027.3207 | 361159.7732 |
| 2001 | DALYs (Disability-Adjusted Life Years) | Observed | 346746.044 | 343730.7662 | 349761.3219 |
| 2002 | DALYs (Disability-Adjusted Life Years) | Observed | 331022.4978 | 328091.3518 | 333953.6438 |
| 2003 | DALYs (Disability-Adjusted Life Years) | Observed | 313331.633 | 310492.588 | 316170.678 |
| 2004 | DALYs (Disability-Adjusted Life Years) | Observed | 301808.971 | 299025.385 | 304592.557 |
| 2005 | DALYs (Disability-Adjusted Life Years) | Observed | 295016.3403 | 292263.7869 | 297768.8937 |
| 2006 | DALYs (Disability-Adjusted Life Years) | Observed | 289633.8318 | 286909.9628 | 292357.7009 |
| 2007 | DALYs (Disability-Adjusted Life Years) | Observed | 284780.2823 | 282080.8506 | 287479.7139 |
| 2008 | DALYs (Disability-Adjusted Life Years) | Observed | 284260.6112 | 281558.907 | 286962.3154 |
| 2009 | DALYs (Disability-Adjusted Life Years) | Observed | 281331.4329 | 278641.1151 | 284021.7506 |
| 2010 | DALYs (Disability-Adjusted Life Years) | Observed | 276572.0963 | 273907.3768 | 279236.8157 |
| 2011 | DALYs (Disability-Adjusted Life Years) | Observed | 272552.7684 | 269905.6278 | 275199.909 |
| 2012 | DALYs (Disability-Adjusted Life Years) | Observed | 270803.7407 | 268162.862 | 273444.6195 |
| 2013 | DALYs (Disability-Adjusted Life Years) | Observed | 271675.0872 | 269029.3312 | 274320.8431 |
| 2014 | DALYs (Disability-Adjusted Life Years) | Observed | 268658.7012 | 266028.148 | 271289.2544 |
| 2015 | DALYs (Disability-Adjusted Life Years) | Observed | 266652.7552 | 264029.8578 | 269275.6527 |
| 2016 | DALYs (Disability-Adjusted Life Years) | Observed | 263054.3344 | 260448.5266 | 265660.1422 |
| 2017 | DALYs (Disability-Adjusted Life Years) | Observed | 255035.0775 | 252469.1594 | 257600.9957 |
| 2018 | DALYs (Disability-Adjusted Life Years) | Observed | 245591.1439 | 243072.4582 | 248109.8296 |
| 2019 | DALYs (Disability-Adjusted Life Years) | Observed | 236151.3275 | 233679.5023 | 238623.1527 |
| 2020 | DALYs (Disability-Adjusted Life Years) | Observed | 225025.3994 | 222608.2226 | 227442.5762 |
| 2021 | DALYs (Disability-Adjusted Life Years) | Observed | 213442.3466 | 211069.5554 | 215815.1378 |
| 2022 | DALYs (Disability-Adjusted Life Years) | Predicted | 213234.8365 | 197017.0956 | 229452.5774 |
| 2023 | DALYs (Disability-Adjusted Life Years) | Predicted | 203474.0061 | 178983.2084 | 227964.8038 |
| 2024 | DALYs (Disability-Adjusted Life Years) | Predicted | 196561.822 | 161957.4399 | 231166.2041 |
| 2025 | DALYs (Disability-Adjusted Life Years) | Predicted | 190182.0047 | 144438.1796 | 235925.8298 |
| 2026 | DALYs (Disability-Adjusted Life Years) | Predicted | 183294.9098 | 125892.438 | 240697.3816 |
| 2027 | DALYs (Disability-Adjusted Life Years) | Predicted | 175585.4607 | 106324.9179 | 244846.0035 |
| 2028 | DALYs (Disability-Adjusted Life Years) | Predicted | 168827.19 | 87060.22134 | 250594.1586 |
| 2029 | DALYs (Disability-Adjusted Life Years) | Predicted | 163885.5685 | 68320.8188 | 259450.3182 |
| 2030 | DALYs (Disability-Adjusted Life Years) | Predicted | 159827.2224 | 49265.58081 | 270388.8641 |
| 2021 | DALYs (Disability-Adjusted Life Years) | Predicted | 213442.3466 | 211069.5554 | 215815.1378 |
